# Supplementary material for: Induced Developmental Arrest of Early Hematopoietic Progenitors Leads to the Generation of Leukocyte Stem Cells
Source: Stem Cell Reports. 2015 Oct 22;5(5):716–27. doi: 10.1016/j.stemcr.2015.09.012 (PMC4649263; doi:10.1016/j.stemcr.2015.09.012)
Supplement: Document S1. Figures S1–S6 and Table S1 [file mmc1.pdf]

Stem Cell Reports, Volume *5*

Supplemental Information

Induced Developmental Arrest of Early Hematopoietic Progenitors Leads to the Generation of Leukocyte Stem Cells

Tomokatsu Ikawa, Kyoko Masuda, Mirelle J.A.J. Huijskens, Rumi Satoh, Kiyokazu Kakugawa, Yasutoshi Agata, Tomohiro Miyai, Wilfred T.V. Germeraad, Yoshimoto Katsura, and Hiroshi Kawamoto

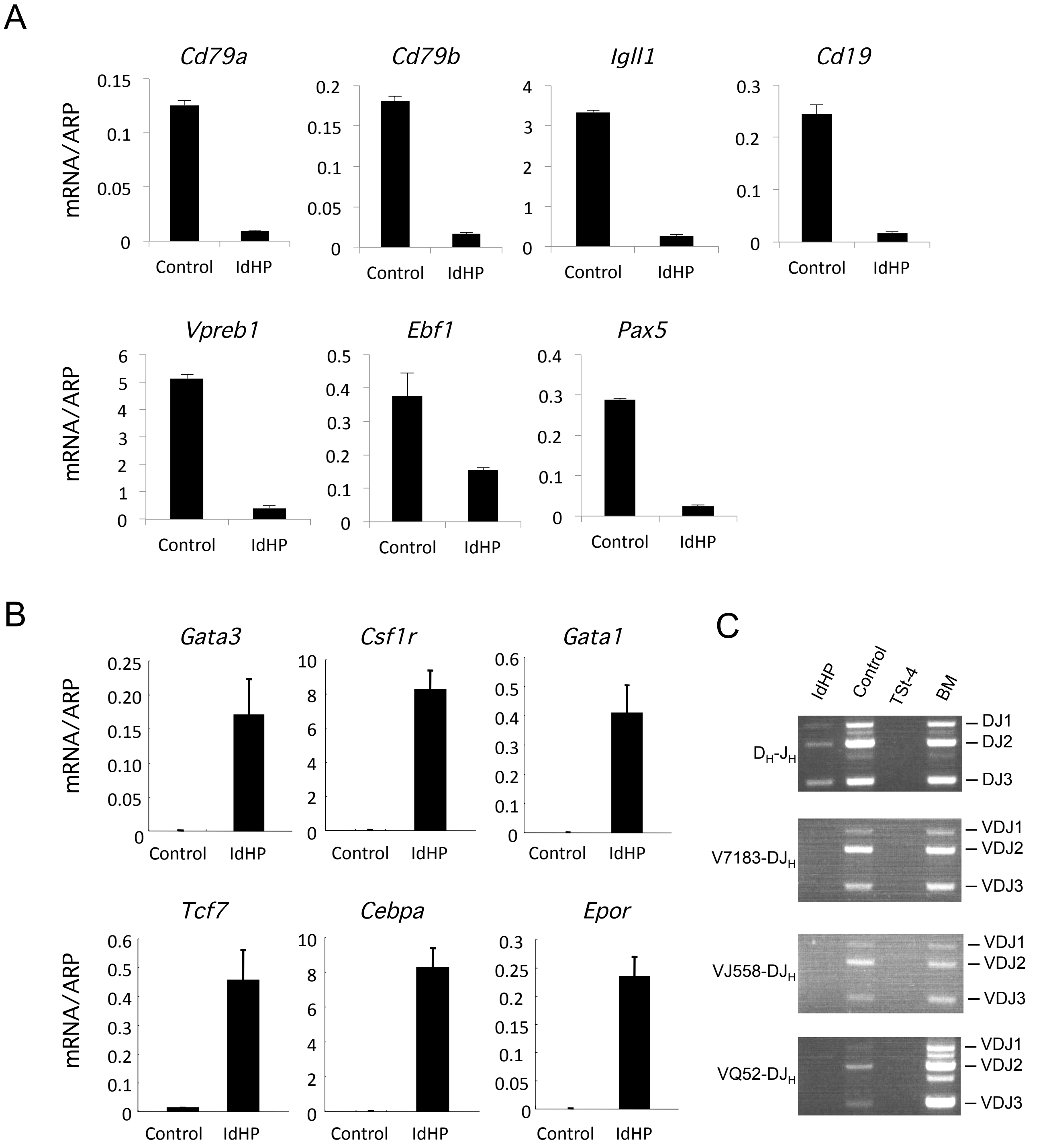

Figure S1. Analysis of gene expression and IgH D-J and V(D)J rearrangements of murine IdHP cells. Related to Figure 1.

(A, B) Quantitative RT-PCR for the indicated genes in IdHP cells as well as Control (Control vector infected) cells is shown. Genes downregulated (A) and upregulated (B) in IdHP cells compared to Control cells are shown. Transcript levels of IdHP cells and Control cells were normalized to the expression of acidic ribosomal protein (ARP) mRNA (n=3). (C) Genomic DNA was isolated from IdHP cells, Control (control vector infected) cells, TSt-4 cells and BM cells and analyzed by PCR for the presence of IgH DJ and V(D)J rearrangements using the indicated primers. Data are shown as mean ± SD from three independent experiments.

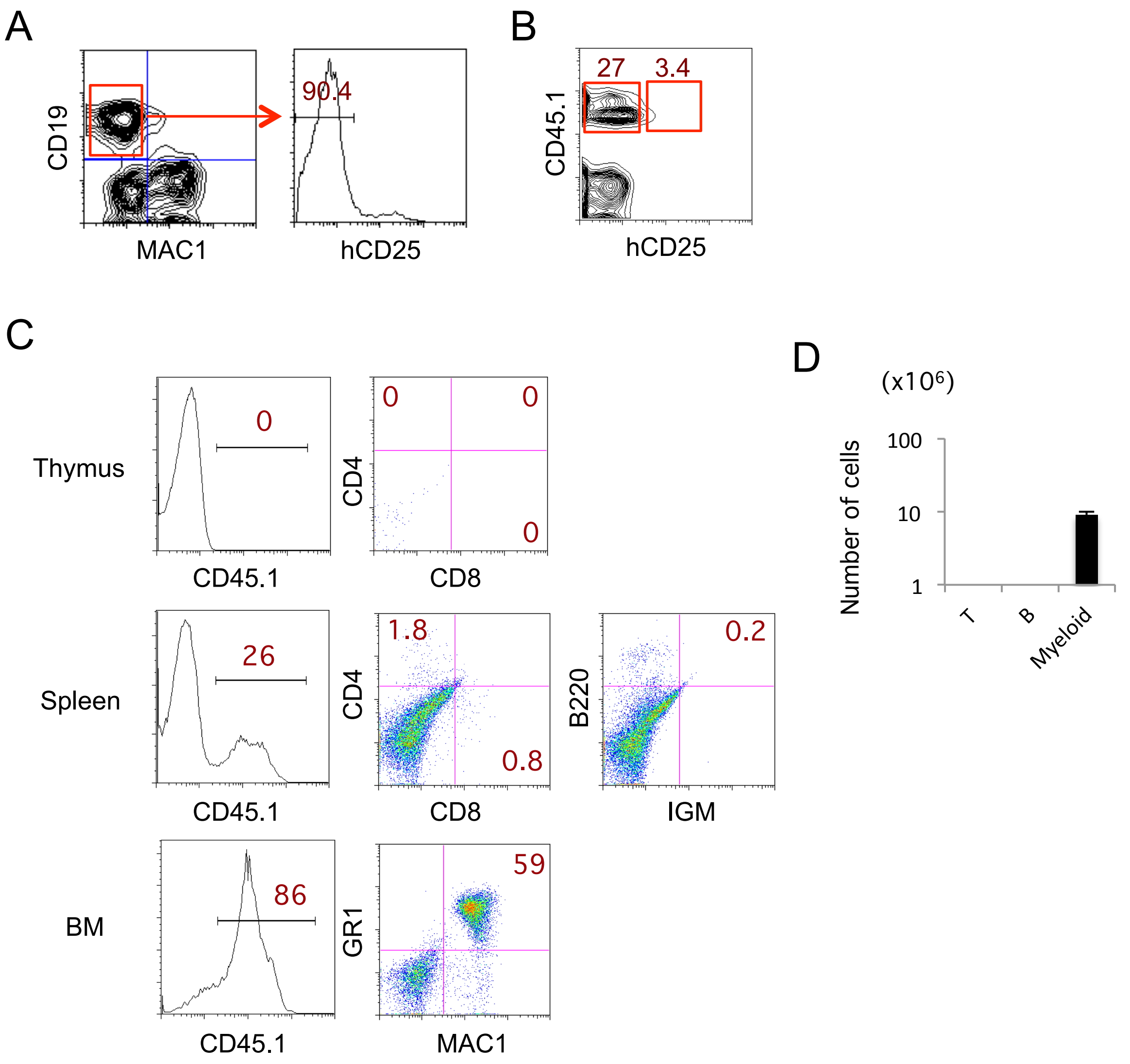

Figure S2. In vivo differentiation potential of IdHP cells. Related to Figure 2.
(A) Flow cytometric analysis of hCD25 expression by $MAC1^{-}CD19^{+}$ cells was performed on the PBMCs of chimeras generated by the injection of IdHP cells into sublethally irradiated RAG1-deficient mice. The mice were analyzed 4 weeks after transplantation. (B) Flow cytometric analysis of hCD25 vs CD45.1 was performed on the PBMCs of chimeras generated by the injection of IdHP cells into sublethally irradiated RAG1-deficient mice. The mice were analyzed 12 weeks after transplantation. (C and D) In vivo generation of myeloid, B and T cells of secondary transferred mice from FL-derived IdHP cells. 1 x 10^6 IdHP cells were intravenously injected into sublethally-irradiated NOG mice. After 7 weeks of injection, the $CD45.1^{+}$ BM cells were sorted and transplanted into sublethally-irradiated NOG mice. The mice were analyzed at 8 weeks after secondary injection. (C) Flow cytometric analysis of cells in thymus, spleen and BM of mice transplanted with $CD45.1^{+}$ BM cells derived from FL-IdHP cells. (D) The number of T ($CD4^{+}CD8^{+}$) cells in thymus, B (IGM^{+}) cells in spleen and Myeloid ($MAC1^{+}GR1^{+}$) cells in BM generated from the IdHP cells. Data are shown as mean ± SD from three independent experiments.

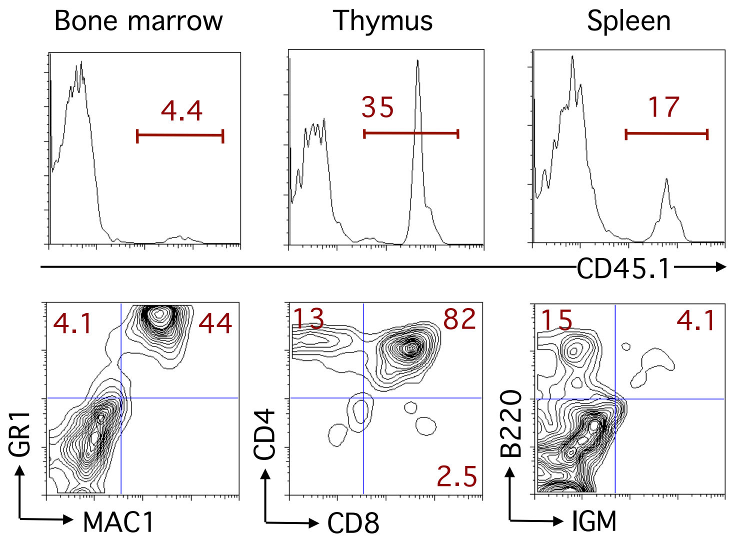

Figure S3. In vivo differentiation potential of T, B and myeloid cells from an IdHP clone. Related to Figure 3.

Generation of lymphoid and myeloid lineage cells in RAG1$^{-/-}$ mice transplanted with cloned IdHP cells. Representative flow cytometric analysis of thymus , spleen, and bone marrow cells in the mice from an IdHP clone (2). The mice were analyzed at 7wks after the transplantation of IdHP clones (n=3).

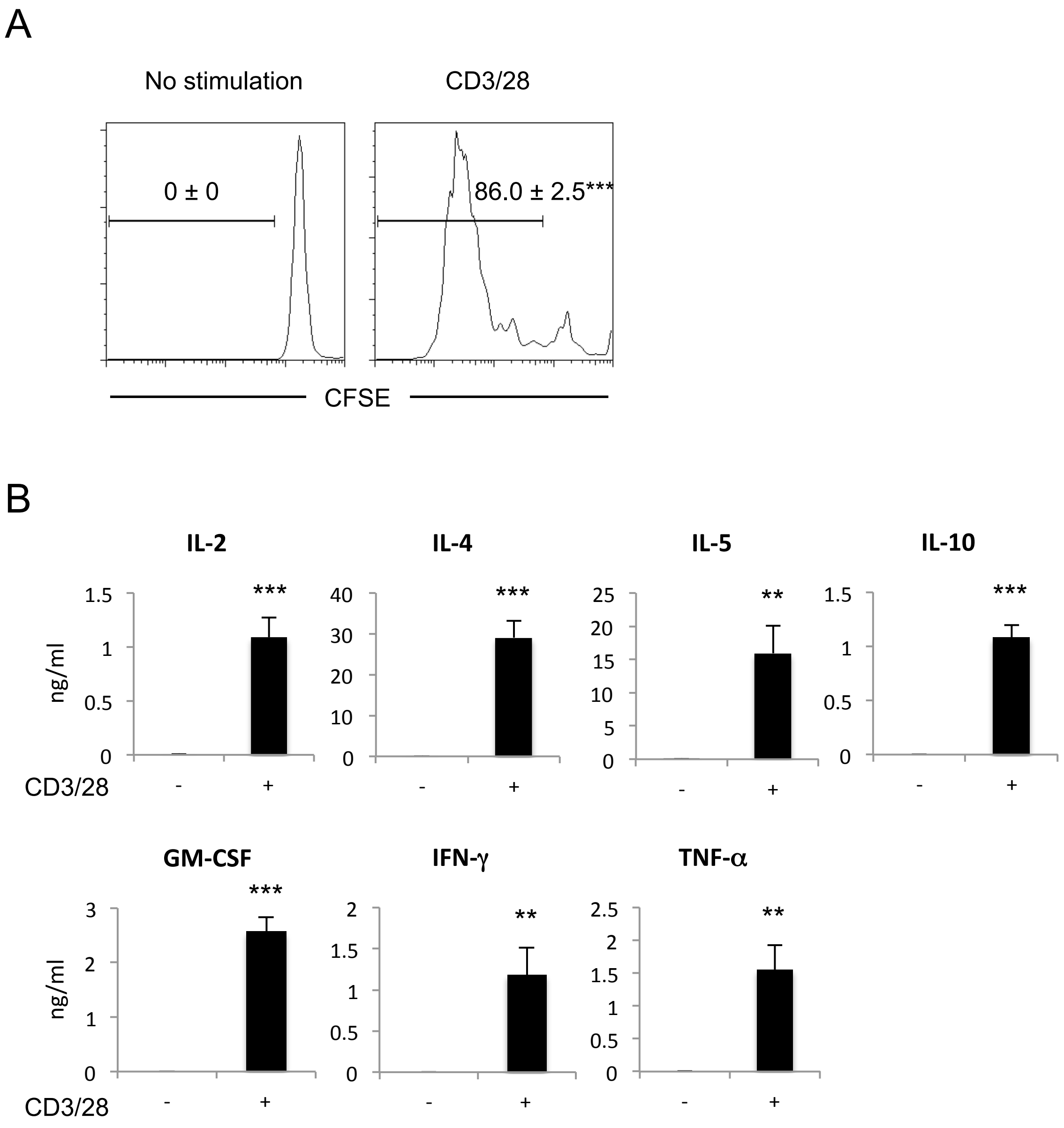

Figure S4. In vitro stimulation of CD4⁺ T cells isolated from spleen of NOG mice generated from BM-derived IdHP cells. Related to Figure 4.

(A) CD4⁺ T cells in spleen generated from BM IdHP cells were sorted and labeled with CFSE. The labeled cells were stimulated with or without plate–coated anti-CD3/28 for 4 days. Flow cytometric analysis of cells after stimulation is shown. (B) Cytokine secretion of the CD4⁺ T cells in response to plate-coated anti-CD3/28 stimulation for 4 days were measured by Bio-Plex analysis. **P<0.01, ***P<0.001. Data are shown as mean ± SD from three independent experiments.

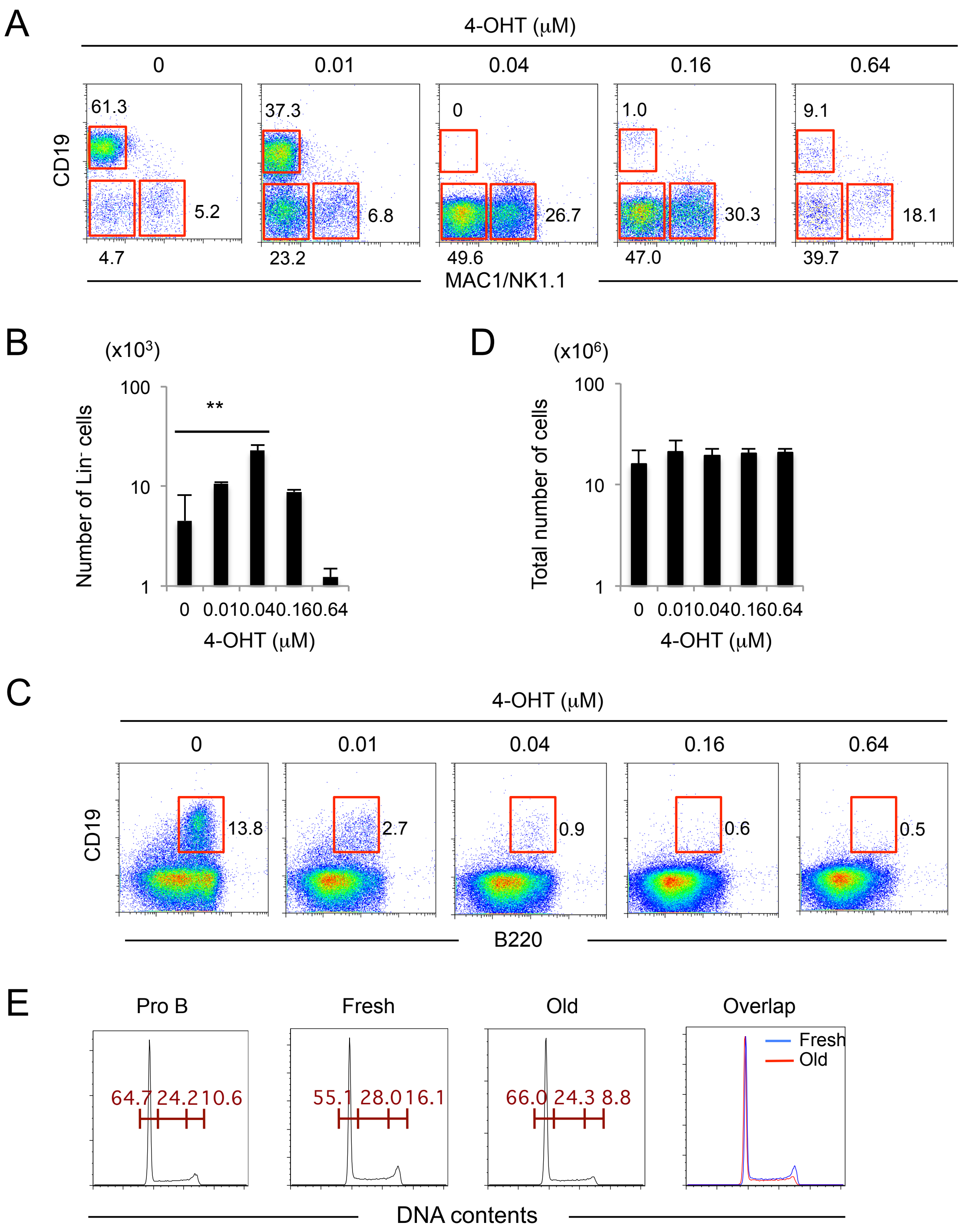

Figure S5. Concentration of 4-OHT in generation and maintenance of Id3-ER transduced cells. Related to Figure 5.

(A) Effect of 4-OHT in generating BM (LKS) progenitors transduced with Id3-ER retrovirus. After transduction, GFP^{+} cells were sorted and cultured on TSt-4 stromal cells supplemented with SCF, IL-7 and FLT3-L at the increasing concentration of 4-OHT for 4weeks. Flow cytometric profiles for CD19 vs. MAC1/NK1.1 are shown. (B) Numbers of MAC1^{-}NK1.1^{-}CD19^{-} (Lin^{-}) cells at the indicated concentration of 4-OHT are shown (n=3). (C) Maintenance of Id3-ER transduced cells at the increasing concentration of 4-OHT. Id3-ER transduced cells (6 x 10^{5} cells) were cultured on TSt-4 cells in the presence of SCF, IL-7 and FLT3-L at the increased concentration of 4-OHT for 2 weeks. The percentage of B220^{+}CD19^{+} cells at the indicated concentration of 4-OHT is shown. (D) Total numbers of cells at the indicated concentration of 4-OHT are shown (n=3). (E) Cell cycle status of Pro B cells, fresh (2 month cultured) IdHP cells, and old (10 months cultured) IdHP cells generated from LKS cells in mouse BM is shown (n=3). ***P*<0.01. Data are shown as mean ± SD from three independent experiments.

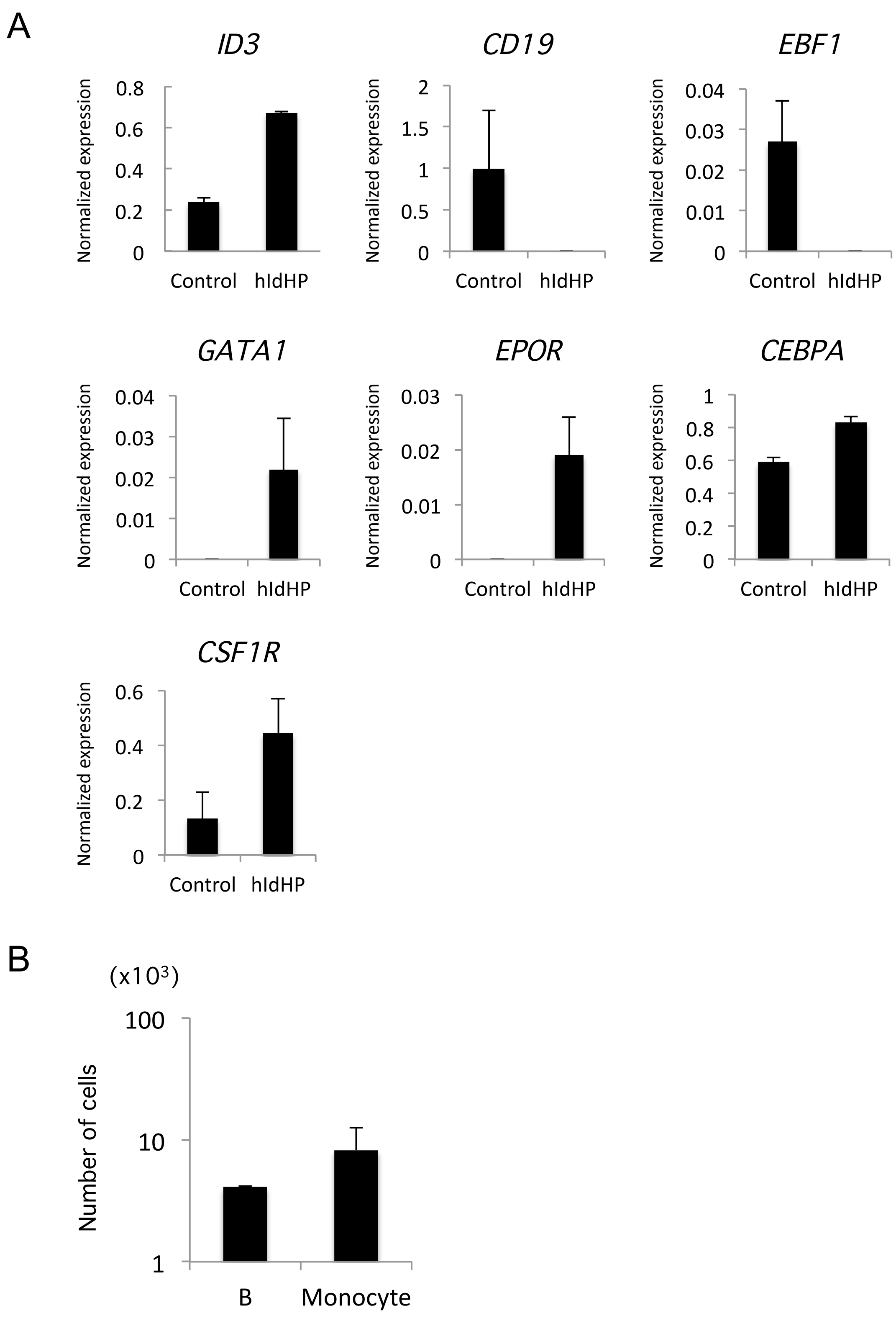

Figure S6. Analysis of gene expression and developmental potential of human IdHP cells. Related to Figure 6

(A) Quantitative RT-PCR for the indicated genes in IdHP cells as well as Control (Control vector infected) cells generated from human CB CD34$^+$ cells is shown (n=3). (B) The number of B (CD33$^-$CD19$^+$) cells and monocytes (CD33$^+$CD14$^+$) in BM generated from human iLS cells were shown (n=3). Flow cytometric profiles are shown in Fig. 6G. Data are shown as mean ± SD from three independent experiments.

Table S1. RT-PCR primers sequences

Gene Name	Forward	Reverse
Cd79a	TATGTCTGACTCCAGCATCC	GGGAAGGACAAGATTAGGTG
Cd79b	CTCTGGGGATAGACTTGACC	GAACCATGGTCCTCCTAGCA
Igll1	GTTCTAATGGGATGCTAGGC	AGCGTCCTTCTCTTATCAGG
Cd19	CAGTGATGGGACTAGCAGAC	GTAGTGTTGCCAGAAACTCG
VpreB1	GAGTGGGAAGGAGAAAAGTC	CCTTCCCATACCAGACTAGC
Ebf1	TGGGTTACAGGTCATATTCG	GAACTGCTTGGACTTGTACG
Pax5	CATTCGGACAAAAGTACAGC	GATGCCACTGATGGAGTATG
Gata3	AGGCAAGATGAGAAAGAGTGCCTC	CTCGACTTACATCCGAACCCGGTA
Csf1r	CTTAATGGCACAAAACAAGG	ACGTCACAGAACAGGACATC
Gata1	ATTCCACAGGTTTCTTTTCC	GTAGTAGGCCAGTGCTGTAG
Tcf7	TGCTGTCTATATCCGCAGGAAG	CGATCTCTCTGGATTTTATTCTCT
Cebpa	CAAGAACAGCAACGAGTACC	GGTDATTGTCACTGGTCAAC
Epor	CCAGCTTTGAGTACACCATC	TCGGACACCACAAGGTATAG
