# Supplementary material for: Induced Developmental Arrest of Early Hematopoietic Progenitors Leads to the Generation of Leukocyte Stem Cells
Source: Stem Cell Reports. 2015 Oct 22;5(5):716–27. doi: 10.1016/j.stemcr.2015.09.012 (PMC4649263; doi:10.1016/j.stemcr.2015.09.012)
Supplement: Document S2. Article plus Supplemental Information [file mmc2.pdf]

Stem Cell Reports
Article

OPEN ACCESS

Induced Developmental Arrest of Early Hematopoietic Progenitors Leads to the Generation of Leukocyte Stem Cells

Tomokatsu Ikawa,[1,2,*] Kyoko Masuda,[2,3] Mirelle J.A.J. Huijskens,[4] Rumi Satoh,[2] Kiyokazu Kakugawa,[2] Yasutoshi Agata,[5] Tomohiro Miyai,[1,6] Wilfred T.V. Germeraad,[4] Yoshimoto Katsura,[2,7] and Hiroshi Kawamoto[2,3,*]

[1]Laboratory for Immune Regeneration, RIKEN Center for Integrative Medical Sciences, Yokohama 230-0045, Japan
[2]Laboratory for Lymphocyte Development, RIKEN Research Center for Allergy and Immunology, Yokohama 230-0045, Japan
[3]Department of Immunology, Institute for Frontier Medical Sciences, Kyoto University, Kyoto 606-8507, Japan
[4]Division of Haematology, Department of Internal Medicine, Maastricht University Medical Centre, PO Box 616, 6220 MD Maastricht, the Netherlands
[5]Department of Immunology and Cell Biology, Graduate School of Medicine, Kyoto University, Kyoto 606-8501, Japan
[6]Graduate School of Frontier Biosciences, Osaka University, Osaka 565-0871, Japan
[7]Division of Cell Regeneration and Transplantation, Advanced Medical Research Center, School of Medicine, Nihon University, Tokyo 173-8610, Japan
*Correspondence: tomokatsu.ikawa@riken.jp (T.I.), kawamoto@frontier.kyoto-u.ac.jp (H.K.)
http://dx.doi.org/10.1016/j.stemcr.2015.09.012
This is an open access article under the CC BY license (http://creativecommons.org/licenses/by/4.0/).

SUMMARY

Self-renewal potential and multipotency are hallmarks of a stem cell. It is generally accepted that acquisition of such stemness requires rejuvenation of somatic cells through reprogramming of their genetic and epigenetic status. We show here that a simple block of cell differentiation is sufficient to induce and maintain stem cells. By overexpression of the transcriptional inhibitor ID3 in murine hematopoietic progenitor cells and cultivation under B cell induction conditions, the cells undergo developmental arrest and enter a self-renewal cycle. These cells can be maintained in vitro almost indefinitely, and the long-term cultured cells exhibit robust multi-lineage reconstitution when transferred into irradiated mice. These cells can be cloned and re-expanded with 50% plating efficiency, indicating that virtually all cells are self-renewing. Equivalent progenitors were produced from human cord blood stem cells, and these will ultimately be useful as a source of cells for immune cell therapy.

INTRODUCTION

Somatic tissues with high turnover rates, such as skin, intestinal epithelium, and hematopoietic cells, are maintained by the activity of self-renewing stem cells, which are present in only limited numbers in each organ (Barker et al., 2012; Copley et al., 2012; Fuchs and Chen, 2013). For example, the frequency of hematopoietic stem cells (HSCs) in the mouse is about 1 in 10^5 of total bone marrow (BM) cells (Spangrude et al., 1988). Once HSCs begin the differentiation process, their progeny cells have hardly any self-renewal capacity, indicating that self-renewal is a special feature endowed only to stem cells.

Cells such as embryonic stem (ES) cells that retain self-renewal potential and multipotency only in vitro can also be included in the category of stem cells. Such stemness of ES cells is thought to be maintained by formation of a core transcriptional network and an epigenetic status unique to ES cells (Lund et al., 2012; Meissner, 2010; Ng and Surani, 2011). A stem cell equivalent to ES cells, called induced pluripotent stem (iPS) cells, can be produced from somatic cells by overexpression of only a few specific transcription factors (OCT3/4, SOX2, KLF4, and C-MYC), which are thought to be the essential components in forming the core network of transcriptional factors that define the status of ES cells (Takahashi et al., 2007; Takahashi and Yamanaka, 2006; Yamanaka, 2012). It is thus generally conceived that acquisition of such a network for a somatic cell depends on the reprogramming of the epigenetic status of that cell.

On the other hand, it could be envisioned that the self-renewing status of cells represents a state in which their further differentiation is inhibited. It is known, for example, that to maintain ES/iPS cells, factors such as leukemia inhibitory factor and basic fibroblast growth factor, for mouse and human cultures, respectively (Williams et al., 1988; Xu et al., 2005), are required, and these factors are thought to block further differentiation of the cells. In this context, it has previously been shown that systemic disruption of transcription factors essential for the B cell lineage, such as PAX5, E2A, and EBF1, leads to the emergence of self-renewing multipotent hematopoietic progenitors, which can be maintained under specific culture conditions (Ikawa et al., 2004a; Nutt et al., 1999; Pongubala et al., 2008). It has recently been shown that the suppression of lymphoid lineage priming promotes the expansion of both mouse and human hematopoietic progenitors (Mercer et al., 2011; van Galen et al., 2014). Therefore, it would seem theoretically possible to make a stem cell by inducing inactivation of these factors at particular developmental stages. Conditional depletion of PAX5 in B cell lineage committed progenitors, as well

as mature B cells, resulted in the generation of T cells from the B lineage cells (Cobaleda et al., 2007; Nutt et al., 1999; Rolink et al., 1999). These studies, however, were mainly focused on the occurrence of cell-fate conversion by dedifferentiation of target cells. Therefore, the minimal requirement for the acquisition of self-renewal potential remains undetermined.

Our ultimate goal is to obtain sufficient number of stem cells by expansion to overcome the limitation of cell numbers for immune therapies. We hypothesize that stem cells can be produced by simply blocking differentiation. As mentioned earlier, self-renewing multipotent progenitors (MPPs) can be produced by culturing E2A-deficient hematopoietic progenitors in B cell-inducing conditions (Ikawa et al., 2004a). Because it remains unclear at which developmental stage the acquisition of self-renewing potential has occurred in the case of such a systemic deletion, we thought to develop a method in which E2A function could be inactivated and reactivated in an inducible manner. We decided to use the ID3 protein for this purpose, because it is known that ID proteins serve as dominant-negative inhibitors of E proteins (Norton et al., 1998; Sayegh et al., 2003). Here we show that the overexpression of ID3 into HSCs or hematopoietic progenitor cells (HPCs) in both mouse and human induces the stemness of the progenitors and that the cells acquire the self-renewal activity. The ID3-expressing cells can be maintained in vitro as MPPs with T, B, and myeloid lineage potentials.

RESULTS

Generation of ID3-Transduced Hematopoietic Progenitors

Murine hematopoietic progenitors isolated as $LIN^-C\text{-}KIT^+SCA\text{-}1^+$ (LKS) cells from the fetal liver (FL) were transduced with a retroviral vector containing Id3 or a control vector, and the transduced cells were cultured under B cell-inducing conditions (Figure 1A). The LKS cells transfected with the control vector differentiated into $CD19^+$ B cells, but the ID3-overexpressing cells showed developmental arrest at the $B220^{low}CD19^-$ stage (Figure 1B). Transcript levels of B lineage-associated genes (e.g., *Cd79a*, *Cd79b*, and *Vpreb1*) in ID3-induced hematopoietic progenitor (IdHP) cells were at least 10-fold lower than those in control cells (Figure S1A). Instead, the IdHP cells prominently express genes associated with other lineages (e.g., *Gata3*, *Gata1*, and *Cebpa*) (Figure S1B). Moreover, only diversity-joining (D-J) but not variable-DJ(V-DJ) of immunoglobulin heavy chain (Igh) gene was detectable in IdHP cells (Figure S1C). These data indicate that the IdHP cells are phenotypically similar to the earliest B cell progenitors, so-called pre-pro B cells, and are almost indistinguishable from the previously reported E2A-deficient MPPs (Ikawa et al., 2004a).

The IdHP cells are relatively large blastic cells, morphologically similar to pre-pro B cells (Figures 1C and 1D), and their gene expression profile is comparable to E2A-deficient progenitors (Figure 1E). The IdHP cells expanded exponentially, i.e., 10^3-fold in 1 month (Figure 1F), and could be maintained for several months or longer. When transferred to culture conditions inductive for myeloid, B, or T lineages, the IdHP cells exhibited the potential to produce all these cell types (Figures 1G–1K); however, erythroid potential was hardly detected, probably because the ID3 suppressed not only E2A activity but all E-protein activities (data not shown).

IdHP Cells Are Multipotent, Maintaining T, B, and Myeloid Lineage Potentials

We then tested the developmental potential of IdHP cells in vivo. A total of 1×10^6 IdHP cells (CD45.1)/mouse were transferred intravenously into sublethally irradiated RAG1-deficient mice (CD45.2) (Figure 2A). After 4 weeks, myeloid cells ($MAC1^+CD19^-$ cells), natural killer (NK) cells ($CD3^-NK1.1^+$ cells), and T cells ($CD3^+NK1.1^-$ cells) were observed in the $CD45.1^+$ fraction in peripheral blood of the recipient mice (Figure 2B). A substantial number of B cells were also detected in these recipients. In these B cells, downregulated expression of the retroviral reporter human CD25 (hCD25) was seen (Figures S2A and S2B), which often occurs with a retrovirally introduced gene. The generation of B cells in vitro shown in Figure 1H could be similarly explained. Such B cell generation indicates that IdHP cells have the potential to produce B cells, a clear difference from E2A-deleted progenitors. T, B, and myeloid cells were generated in the thymus, spleen, and BM of mice reconstituted with the IdHP cells at 7 weeks of transplantation (Figures 2C and 2D). The $CD4^+$ T cells in the spleen generated from the IdHP cells normally proliferated upon anti-CD3/28 stimulation (Figure 2E), confirming the multilineage differentiation potential and functionality of the IdHP cells. To test whether the IdHP cells exhibit self-renewal activity in vivo, the BM cells in NOD/Shi-*scid*, $IL2R\gamma^{null}$ (NOG) mice derived from IdHP cells were further transferred into NOG mice. The mice were analyzed at 8 weeks of secondary transplantation. The results demonstrated that the IdHP cells derived from BM cells exhibited only residual myeloid, but not T and B cell potential (Figures S2C and S2D). The data ruled out the possibility that the IdHP cells might contain MPPs that acquired self-renewal activity or ID3-induced HSC property in vivo.

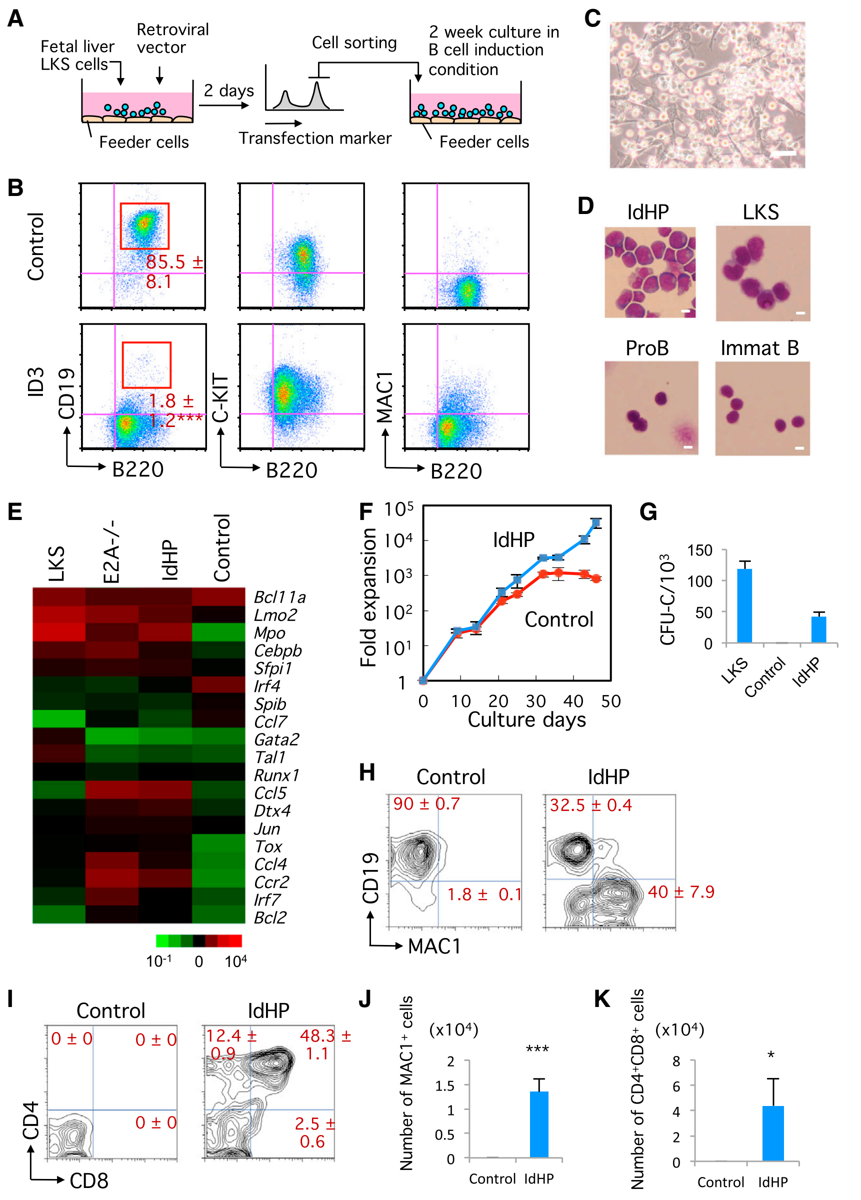

Figure 1. Generation of IdHP Cells from Murine HSCs or HPCs

(A) Schematic representation of IdHP cell generation.
(B) Flow cytometric analysis of control (empty vector) and ID3-overexpressing FL progenitor cells (n = 3).
(C) Photomicrograph of IdHP cells. Scale bar, 10 μm.
(D) Wright's staining of IdHP cells, LKS cells, pro B cells, and immature B (Immat B) cells from BM. Scale bars, 10 μm.
(E) Microarray analysis of gene expression in LKS cells, E2A$^{-/-}$ HPCs, IdHP cells, and pro B cells derived from cultures of control vector-expressing FL progenitors.
(F) In vitro expansion of IdHP and control cells. Viable cells were counted at an each time point (n = 3).
(G) CFU-C assay of LKS, control, and IdHP cells (n = 3).
(H) Myeloid and B cell generation from IdHP cells in vitro. Flow cytometric profiles of IdHP cells cultured on TSt-4 stromal cells for 14 days are shown (n = 3).
(I) T cell generation from IdHP cells in vitro. Flow cytometric profiles of control and IdHP cells cultured on TSt-4/DLL1 stromal cells for 12 days are shown (n = 3).
(J) The number of MAC1$^+$ cells generated from IdHP and control cells on TSt-4 stromal cells is shown (n = 3). The FACS profile from IdHP cells is shown in (H).
(K) The number of CD4$^+$CD8$^+$ cells generated from IdHP cells on TSt-4/DLL1 stromal cells is shown (n = 3). The FACS profiles are shown in (H) and (I). Student's t test, $^*p < 0.05$, $^{***}p < 0.001$. Data are shown as mean ± SD from three independent experiments.
See also Figure S1.

IdHP Cells Are Multipotent at a Clonal Level

To examine whether IdHP cells retain self-renewal potential, 96 IdHP cells were individually seeded and cultured in the same conditions (Figure 3A). Each clone was expanded with a plating efficiency of 50%. All clones were able to proliferate unlimitedly like IdHP cells as long as the cells were properly cultured. Three randomly selected clones were further expanded, and the cells (1×10^6 cells/mouse) were subsequently transferred into sublethally irradiated recipient mice. After 4 weeks, reconstitution of myeloid cells, B cells, NK cells, and T cells derived from the transferred cells was observed in the peripheral blood of these recipients (Figure 3B). Mice transplanted with clone 1 cells were sacrificed 8 weeks after the transfer, and cells from the thymus, spleen, and BM were analyzed. In the BM, MAC1$^+$GR1$^+$ myeloid cells were observed among the CD45.1$^+$ cells (Figures 3C and 3D). CD45.1$^+$ CD4$^+$CD8$^+$DP cells, as well as CD4SP and CD8SP cells, were seen in the thymus, and CD45.1$^+$B220$^+$IGM$^+$ mature B cells were found in the spleen (Figures 3C and 3D). These data indicate that the production of several lineages of cells is ongoing in recipient mice. Because IdHP cells are originally derived from pre-pro B stage cells, they usually bear at least a single allele of D_H-J_H rearrangements of the *Igh* genes. Clone 1 and 2 had a rearrangement involving the J_H3 gene segment, and all lineage of cells from various

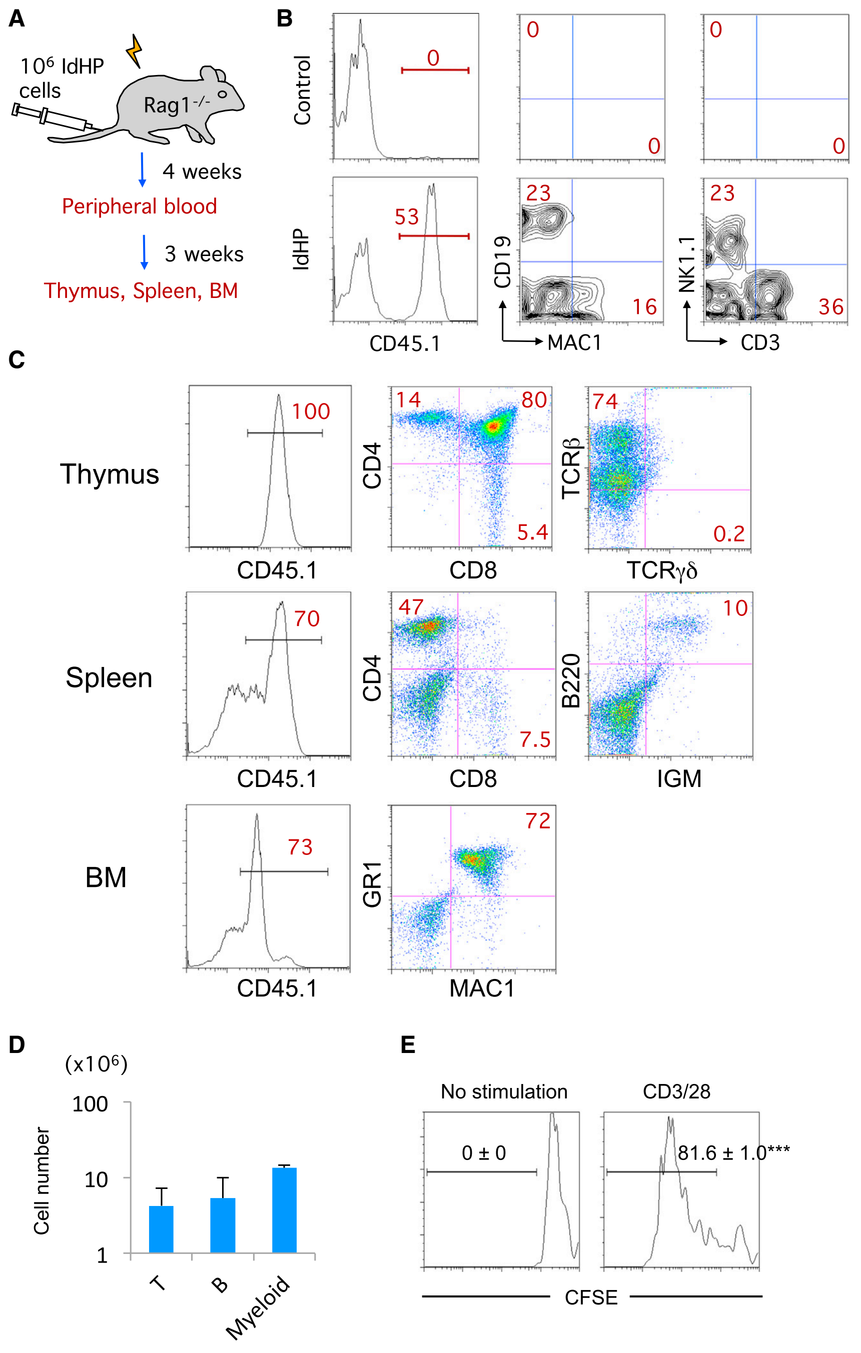

Figure 2. In Vivo Generation of Myeloid, B, and T Cells from IdHP Cells

(A) Schematic representation of the in vivo model for investigating the developmental potential of IdHP cells.

(B) Flow cytometric analysis of cells in the peripheral blood of mice transplanted with control or IdHP cells 4 weeks after injection. Donor derived PBMCs ($CD45.1^+$) were analyzed for the expression of MAC1 versus CD19 and CD3 versus NK1.1.

(C) Flow cytometric analysis of cells in thymus, spleen, and BM of mice transplanted with IdHP cells 7 weeks after injection.

(D) The number of T ($CD4^+CD8^+$) cells in thymus, B (IGM^+) cells in spleen, and myeloid ($MAC1^+GR1^+$) cells in BM generated from the IdHP cells.

(E) $CD4^+$T cells in spleen generated from the IdHP cells were sorted and labeled with CFSE. The labeled cells were stimulated with plate-coated anti-CD3/28 for 4 days. Flow cytometric analysis of the cells after stimulation is shown (n = 3). ***$p < 0.001$. Data are shown as mean ± SD from three independent experiments.

See also Figure S2.

tissues in recipient animals transferred with clone 1 and 2 cells had the same rearrangement (Figures 3E and S3), indicating that all progeny cells were derived from clone 1 and 2, respectively. These results indicate that the IdHP cells were multipotent at the clonal level.

Generation of IdHP Cells from Mouse BM

To determine whether IdHP cells can also be generated from adult BM progenitors, the LKS cells in the BM of B6CD45.1 mice were transduced with a retroviral vector containing ID3 and the transduced cells were cultured under B cell-inducing conditions. The IdHP cells were generated in 1 month, just like the FL-derived IdHP cells (Figure 4A). The BM-derived IdHP cells exponentially expanded and could be maintained for several months or longer, similar to the FL-IdHP cells. To determine the developmental potential of the BM-IdHP cells in vivo, 1×10^6 IdHP cells/mouse were transplanted into

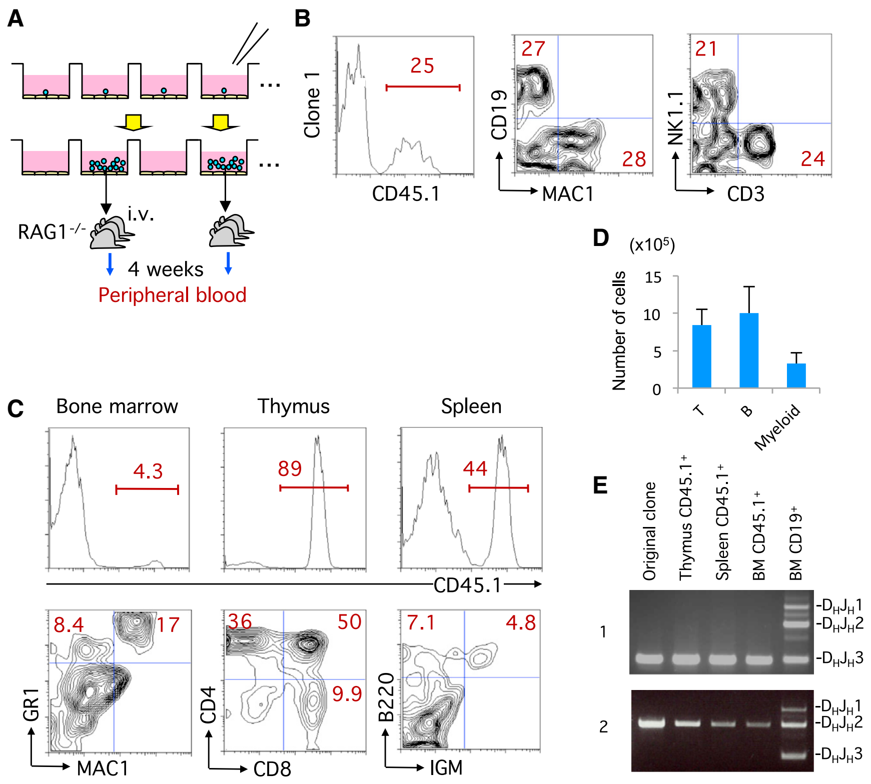

Figure 3. Lymphoid-Myeloid Lineage Potential of Single IdHP Cells

(A) Schematic representation of cloning of IdHP cells and analysis of the developmental potential of individual IdHP clones.

(B) Generation of lymphoid and myeloid lineage cells in RAG1$^{-/-}$ recipients transplanted with cloned IdHP cells. Flow cytometric profiles of donor-type (CD45.1$^+$) PBMCs 4 weeks after transplantation are shown.

(C) Analysis of thymus, spleen, and bone marrow cells in the mice generated in the experiment shown in (B).

(D) The number of T (CD4$^+$CD8$^+$) cells in thymus, B (IgM$^+$) cells in spleen, and myeloid (MAC1$^+$GR1$^+$) cells in BM of RAG1$^{-/-}$ recipients generated in the experiment (C). Data are shown as mean ± SD from three independent experiments (n = 3).

(E) Analysis of *Igh* D-J rearrangement in the donor-type (CD45.1$^+$) cells of the thymus, spleen, and bone marrow of mice used in experiment (C, and Figure S3) (n = 3).

See also Figure S3.

sublethally irradiated immunodeficient (NOG) mice. After 7 weeks, thymic populations, as well as MAC1$^+$GR1$^+$ myeloid cells in the BM, were found to be nicely reconstituted (Figures 4B and 4C). The CD4$^+$ T cells in the spleen generated from BM-IdHP cells proliferated and secreted various cytokines in response to anti-CD3/28 stimulation in vitro (Figure S4).

To determine from which stage IdHP cells can be generated, we transfected CD34$^-$LKS (HSC), CD34$^+$LKS (MPP), common lymphoid progenitor (CLP), and common myeloid progenitor (CMP) cells from the BM with ID3 and control retroviruses. The transfected cells were cultured on TSt-4 stromal cells in the presence of stem cell factor (SCF), interleukin-7 (IL-7), and FMS-like tyrosine kinase 3 ligand (FLT3-L) in a same manner as IdHP cells from LKS cells. While virtually all control cells from HSC, MPP, and CLP cells became CD19$^+$ cells after 2 weeks of culture, the control cells from CMP became myeloid cells (Figure 4D). Id3-transduced cells from HSC and MPP, but not from other types of progenitors, exhibited similar expansions as IdHP cells from LKS cells. The finding that MPP cells can generate IdHP cells may indicate that the self-renewal activity can be endowed to a non-self-renewing progenitor by ectopic expression of ID3. The failure in inducing IdHP cells from CLP cells does not necessarily mean that self-renewal activity is acquired before the CLP stage, because expression of ID3 will take some time after transfection. These data indicate that the self-renewing IdHP cells with developmental potential and functionality similar to those of the FL-IdHP cells can be generated from adult BM.

Generation of Inducible IdHP Cells Using ID3-ER Retrovirus

To examine whether IdHP cells are really arrested early in B cell development, we used the ID3 protein fused with estrogen receptor, ID3-ER, a more controllable system for the expression of ID3 in which ID3-ER protein normally resides in the cytoplasm but goes into the nucleus and functions as a transcriptional inhibitor for E proteins only when 4-hydroxytamoxifen (4-OHT) is added (Sayegh et al., 2003). LKS cells from the FL of B6CD45.1 mice that had been transduced with the ID3-ER retrovirus and cultured in the presence of 4-OHT showed a similar developmental arrest and entered a self-renewal cycle similar to that of IdHP cells (Figures 5A–5C and S5A–S5D). Removal of 4-OHT did not have any impact on cell growth for at least 10 days, but virtually all cells became CD19$^+$ within 7 days (Figure 5D), indicating that arrested cells restarted differentiation toward B cells upon removal of 4-OHT. These results indicate that IdHP cells represent cells differentiating toward the B cell lineage but are arrested just before the B cell lineage

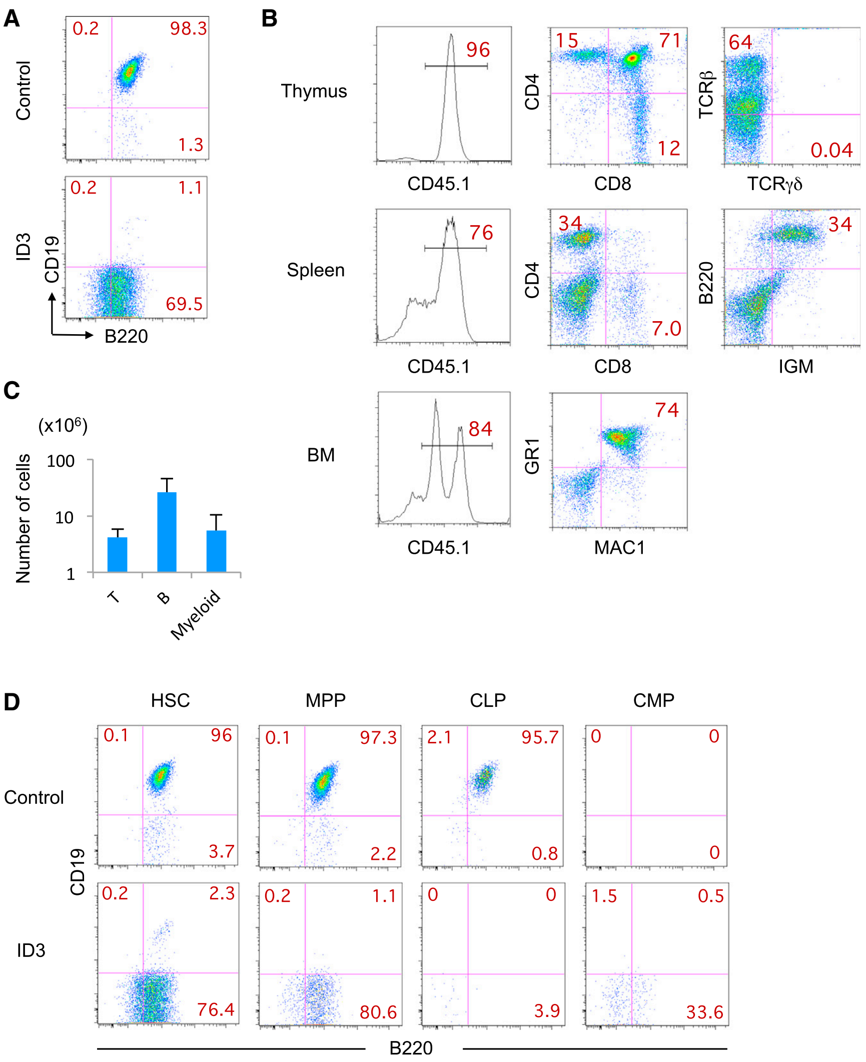

Figure 4. In Vivo Generation of Myeloid, B, and T Cells from BM-Derived IdHP Cells

(A) ID3 and control retroviruses were infected with LKS cells in BM of B6CD45.1 mice, and the infected cells were sorted and cultured on TSt-4 stromal cells in the presence of SCF, IL-7, and FLT3-L for 2 weeks. Flow cytometric profiles of CD19 versus B220 gated on $MAC1^-GR1^-NK1.1^-$ cells are shown.

(B) BM IdHP cells (1×10^6) were intravenously injected into sublethally irradiated NOG mice. Flow cytometric analysis of cells in thymus, spleen, and BM of mice transplanted with BM IdHP cells 7 weeks after injection is shown.

(C) The number of T ($CD4^+CD8^+$) cells in thymus, B (IGM^+) cells in spleen, and myeloid ($MAC1^+GR1^+$) cells in BM generated from the IdHP cells (n = 3) is shown. Data are shown as mean ± SD from three independent experiments (n = 3).

(D) Id3 and control viruses were infected with HSC ($CD34^-LKS$), MPP ($CD34^+LKS$), CLP ($LIN^-C\text{-}KIT^+IL7R^+$), and CMP ($LIN^-C\text{-}KIT^+SCA\text{-}1^-CD16/32^-CD34^+$) cells in BM of B6CD45.1 mice, and the infected cells were sorted and cultured on TSt-4 stromal cells in the presence of SCF, IL-7, and FLT3-L for 2 weeks. Flow cytometric profiles of CD19 versus B220 gated on $MAC1^-GR1^-NK1.1^-$ cells are shown.

See also Figure S4.

determination step, waiting for appropriate developmental cues.

To determine the stability of the ID3-ER-transduced HPCs, the cell-cycle status of fresh (2-month cultured) and old (10-month cultured) progenitor cells were analyzed. Although the proportion of cells at G1, S, and G2/M phases in fresh and old IdHP cells was slightly different, two histograms showed almost complete overlap (Figure S5E). These results indicate that the ID3-ER-transduced progenitor cells are stable and can be maintained without any phenotypic changes for several months or longer.

Thus, by definition, IdHP cells satisfy the criteria of stem cells, which are restricted to the production of leukocytes. We therefore designate these cells as iLS (induced leukocyte stem) cells as a more general term. The iLS cells are not reprogrammed or de-differentiated but only developmentally arrested. Therefore, we propose that the blockage of differentiation due to the absence of developmental cues is sufficient to make stem cells.

Generation of IdHP Cells from Human Cord Blood Hematopoietic Progenitors

Self-renewing progenitor cells whose development can be controlled are a potential source for human immune cell therapy. We therefore attempted to produce human iLS cells. $CD34^+$ cord blood (CB) cells were transduced with a retrovirus encoding the human *ID3* gene and cultured under B cell-inducing conditions. In the control vector group, cells differentiated into $CD19^+$ B cells, although cells expressing the myeloid marker CD33 were also generated under this condition (Figure 6A). In the ID3 overexpression group, cells exhibited higher forward or side scatter properties (Figure 6A), with a larger size and more cytoplasm (Figure 6B). Just like murine iLS cells, human iLS cells showed exponential growth for several

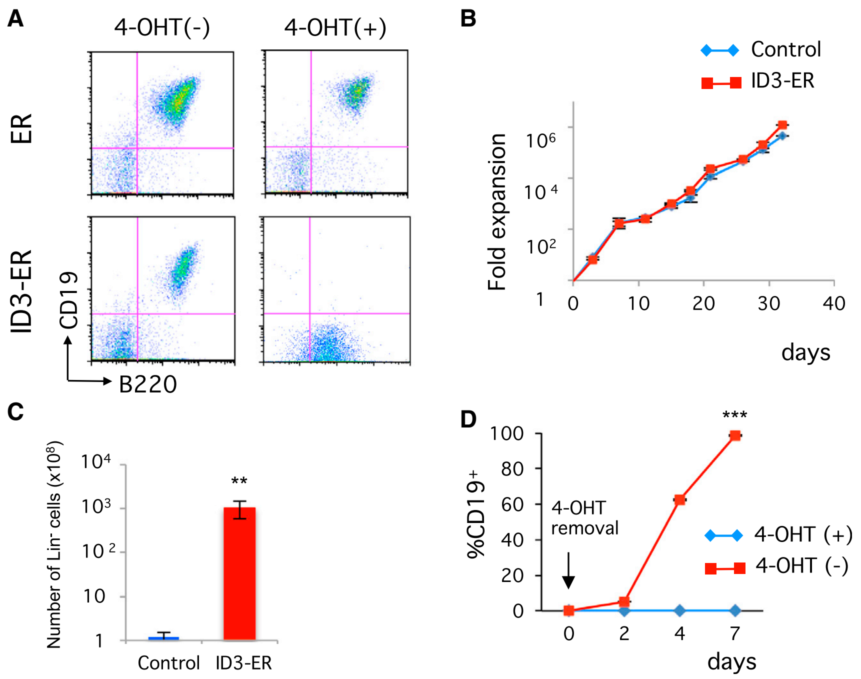

Figure 5. Inducible Generation of IdHP Cells Using an ID3-ER Retrovirus

(A) Developmental potential of FL (LKS) progenitors transduced with control (ER) and ID3-ER retrovirus. After transduction, GFP^+ cells were sorted and cultured on TSt-4 stromal cells supplemented with SCF, IL-7, and FLT3-L in the presence or absence of 4-OHT for 4 weeks. Representative flow cytometric profiles for CD19 versus B220 are shown.

(B) Expansion of FL (LKS) progenitors transduced with control and ID3-ER retrovirus in the presence of 4-OHT. Viable cells were counted at the indicated time points.

(C) Number of LIN^- cells from control and ID3-ER infected cells after 32 days of culture.

(D) B cell generation from ID3-ER-transduced cells after withdrawal of 4-OHT. The percentage of $CD19^+$ cells at the indicated time points is shown. $^{**}p < 0.01$, $^{***}p < 0.001$. Data are shown as mean ± SD from three independent experiments.

See also Figure S5.

weeks or longer (Figure 6C), although with a slower growth rate than that of the mouse case. In addition, human iLS cells exhibited a gene expression pattern similar to that of murine iLS cells (Figures S1A and S6A). These human iLS cells retained the potential to give rise to NK cells and dendritic cells (DCs) or macrophage-like cells in vitro (Figures 6D and 6E). When transferred into sublethally irradiated NOG mice (Figure 6F), human iLS cells gave rise to B cells ($CD19^+CD33^-$) and monocytes ($CD33^+CD14^+$) in the BM of the reconstituted mice (Figures 6G and S6B).

DISCUSSION

Identification of cellular and molecular events regulating self-renewal or differentiation of the cells is a fundamental issue in the stem cell biology or developmental biology field. In the present study, we revealed that the simple inhibition of differentiation in HSCs or HPCs by overexpressing ID proteins and culturing them in suitable conditions induced the self-renewal of hematopoietic progenitors and allowed the extensive expansion of the multipotent cells. The reduction of ID proteins in MPPs resulted in the differentiation of the cells into lymphoid and myeloid lineage cells. Thus, it is possible to manipulate the cell fate by regulating E-protein or ID-protein activities. This inducible system will be a useful tool to figure out the genetic and epigenetic program controlling the self-renewal activity of multipotent stem cells.

Previous studies have shown that hematopoietic progenitors deficient for E2A, EBF1, and PAX5 maintain multilineage differentiation potential without losing their self-renewing capacity (Ikawa et al., 2004a; Nutt et al., 1999; Pongubala et al., 2008), indicating that the inhibition of the differentiation pathway at certain developmental stages leads to the expansion of multipotent stem cells. However, the MPPs were not able to differentiate into B cells because they lacked the activities of transcription factors essential for the initiation of the B lineage program. In addition, a restriction point regulating the lineage-specific patterns of gene expression during B cell specification remained to be determined because of the lack of an inducible system that regulates B cell differentiation. Here we have established the multipotent iLS cells using ID3-ER retrovirus, which can be maintained and differentiated into B cells in an inducible manner by simply adding or withdrawing 4-OHT. The data indicated the essential role of E2A for initiation of the B cell program that restricts other lineage potentials, because the depletion of 4-OHT from the culture immediately leads to the activation of E proteins, such as E2A, HEB, and E2-2, that promote B cell differentiation. This strategy is useful in understanding the cues regulating the self-renewal or differentiation of uncommitted progenitors to the B cell pathway. Analysis of genome-wide gene expression patterns and histone modifications will determine the exact mechanisms that underlie the B cell commitment process.

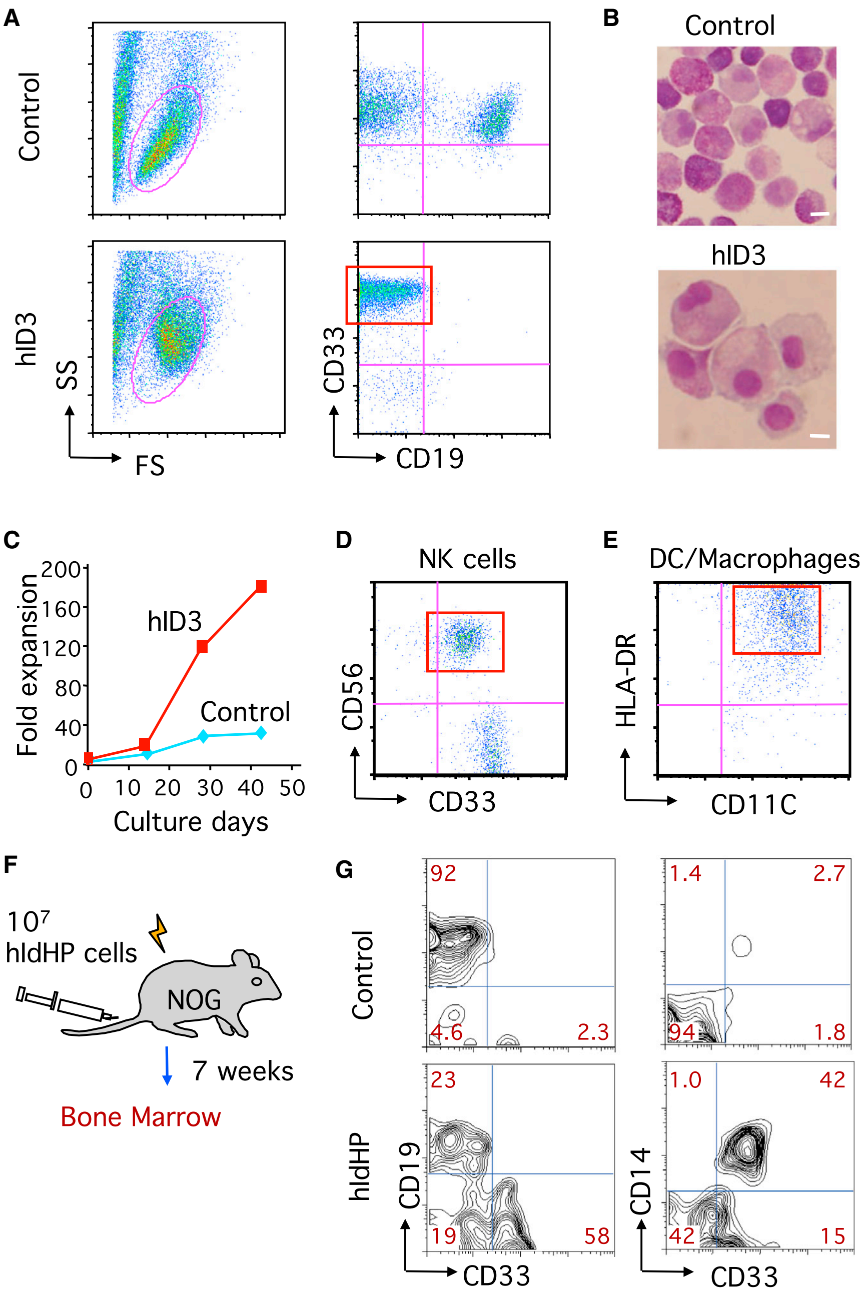

Figure 6. Generation of IdHP Cells from Human CB HSCs

(A) Flow cytometric analysis of cells derived from $CD34^+$ human CB cells transduced with control and human ID3 retrovirus. After transduction, GFP^+ cells were sorted and cultured on TSt-4 stromal cells in the presence of SCF, IL-7, FLT3-L, and thrombopoietin (TPO) for 4 weeks.

(B) Wright's stain of hIdHP and control cells. Scale bars, 10 μm.

(C) Expansion of hIdHP and control cells on TSt-4 stromal cells in the presence of human SCF, IL-7, FLT3-L, and TPO. Viable cells were counted at the indicated time points (n = 3).

(D and E) NK cell (D) and DC (E) generation from hIdHP cells in vitro. Flow cytometric analysis is shown.

(F and G) Generation of $CD19^+$ and $CD14^+$ cells from hIdHP cells in NOG mice. (F) Schematic representation of the examination of the developmental potential of hIdHP cells. (G) Flow cytometric profiles for CD33 versus CD19 and CD33 versus CD14 of human $CD45^+$ cell in BM from transplanted mice. Data are shown from three independent experiments.

See also Figure S6.

The iLS cells can also be generated from human CB hematopoietic progenitors. Human iLS cells exhibited differentiation potential and self-renewal activity similar to those of murine iLS cells, suggesting a similar developmental program during human B cell fate specification. Our data are consistent with a study demonstrating the critical role of the activity of ID and E proteins for controlling the status of human HSCs and progenitors (van Galen et al., 2014). This study reported that the overexpression of ID2 in human CB HSCs enhanced the myeloid and stem cell program at the expense of lymphoid commitment. Specifically, ID2 overexpression resulted in a 10-fold expansion of HSCs, suggesting that the inhibition of E-protein activities promotes the self-renewal of HSCs by antagonizing the differentiation. This raises a question about the functional differences between ID2 and ID3. Id3 seems to suppress the B cell program and promote the myeloid program more efficiently than does ID2, because the ID2-expressing HPCs appear to retain more B cell potential than ID3-expressing iLS

cells (Mercer et al., 2011; van Galen et al., 2014). The self-renewal activity and differentiation potential of ID2-HPCs derived from murine HSCs in the BM seemed to be limited both in vivo and in vitro analysis (Mercer et al., 2011). In our study, the iLS cells retained more myeloid potential and self-renewal capacity during the culture. Strikingly, the multipotent iLS cells enormously proliferated (>10^3-fold in 1 month) as long as the cells were cultured in undifferentiated conditions. This could be due to the functional differences among *Id* family genes. Alternatively, combination with additional environmental signals, such as cytokines or chemokines, may affect the functional differences of ID proteins, although any ID proteins can repress the activation of the E2A targets, such as *Ebf1* and *Foxo1*, that are essential for B cell differentiation. ID1 and ID3, but not ID2, are demonstrated to be negative regulators of the generation of hematopoietic progenitors from human pluripotent stem cells (Hong et al., 2011). Further analysis is required to determine the physiological role of ID proteins in regulating hematopoietic cell fate. It also remains to be determined whether the ID3-ER system can be applied to human progenitors. It would be informative to compare the regulatory networks that control B cell differentiation in mouse and human.

Immune cell therapy has become a major field of interest in the last two decades. However, the required high cell numbers restrain the application and success of immune reconstitution or anti-cancer treatment. For example, DCs are already being used in cell therapy against tumors. One of the major limitations of DC vaccine therapy is the difficulty in obtaining sufficient cell numbers, because DCs do not proliferate in the currently used systems. The method of making iLS cells could be applied to such cell therapies. Taken together, the simplicity of this method and the high expansion rate and retention of multilineage potential of the cells make this cell source appealing for regenerative medicine or immune cell therapy.

In summary, we showed that an artificially induced block of differentiation in uncommitted progenitors is sufficient to produce multipotent stem cells that retain self-renewal activity. Once the differentiation block is released, the cells start differentiating into mature cells both in vivo and in vitro. Thus, this method could be applicable for establishing somatic stem cells from other organs in a similar manner, which would be quite useful for regenerative medicine. The relative ease of making stem cells leads us to conceive that a block in differentiation is essential not only in other types of artificially engineered stem cells, such as ES cells and iPS cells, but also in any type of physiological somatic stem cell. In this context, it is tempting to speculate that it could have been easy for a multicellular organism to establish somatic stem cells by this mechanism during evolution.

EXPERIMENTAL PROCEDURES

Mice

C57BL/6 (B6) and B6CD45.1 mice were purchased from CLEA Japan. NOD/Shi-*scid*, IL2Rγ^{null} (NOG) mice were purchased from the Central Institute for Experimental Animals. We used 6- to 8-week-old female mice for the transfer experiments. Embryos at various stages of gestation were obtained from timed pregnancies. The day of observing the vaginal plug was designated as 0 dpc. Animal and human experiments were approved by the RIKEN Institutional Animal Care and Use and Human Subjects Use Committees, respectively.

Antibodies

The following antibodies were purchased from BD Biosciences: fluorescein isothiocyanate (FITC)-conjugated erythroid lineage cells (TER119: 561032), MAC1 (553310), GR1 (553127), CD11C (557400), B220 (553088), THY1.2 (553004), CD8A (553031), CD4 (553651), NK1.1 (553164), CD3ε (553062), CD19 (553785), TCRγδ (553177), phycoerythrin (PE)-conjugated SCA-1 (553336), CD4 (553653), CD19 (553786), GR1 (553128), NK1.1 (553165), TCRβ (553172), human CD11C (347637), human CD25 (555432), human CD33 (555450), allophycocyanin (APC)-conjugated CD45.1 (558701), CD45.2 (558702), C-KIT (553556), CD19 (550992), human CD19 (555415), human CD56 (555518) and human HLA-DR (559866). FITC-IGM (11-5790-81) was purchased from eBioscience. TER119, MAC1, GR1, B220, CD19, NK1.1, CD3ε, CD4, and CD8A were used as Lin markers.

Growth Factors

Recombinant murine (rm) SCF, IL-1α, IL-3, IL-7, IL-15, FLT3-L, G-CSF, M-CSF, and GM-CSF and recombinant human (rh) SCF, IL-7, IL-15, FLT3-L, GM-CSF and TNFα were purchased from R&D Systems.

Isolation of Hematopoietic Progenitors

Single cell suspensions of FL cells from 13 to 15 dpc embryos or BM cells of B6CD45.1 mice were prepared as described previously (Ikawa et al., 2004b). Cells were then incubated with mAbs specific for anti-lineage markers (TER119, MAC1, GR1, B220, and THY1.2) for 20 min on ice. Lin$^+$ cells were depleted with Dynabeads Sheep anti-Rat IgG (Invitrogen) according to the manufacturer's protocol. The LIN$^-$ cells were used for cell sorting. The procedure for isolating LIN$^-$SCA-1$^+$C-KIT$^+$ (LKS) populations from the FL and BM and isolating pro B cells (IGM$^-$B220$^+$CD19$^+$CD43$^+$) and immature B (IGM$^+$B220$^+$CD19$^+$) cells from BM has been described elsewhere (Ikawa et al., 2004a, 2004b).

Retroviral Constructs, Viral Supernatants, and Transduction

The TAC retroviral vector (pCSretTAC) is based on the S-001 retrovirus construct (obtained from H. Spits) and was generated by

replacing the coding sequence of EGFP with the human *IL2RA* gene encoding CD25. The full-length cDNA for murine *Id3* was cloned into the pCSretTAC vector (mID3-TAC). The ID3-ERT2 fusion construct containing the full-length cDNA for human *ID3* fused to the mutated ligand-binding domain of the human estrogen receptor (ERT2) was cloned into the pMCS retrovirus vector (a gift from T. Kitamura). The human *ID3* cDNA was cloned into the pMX retrovirus construct (obtained from T. Kitamura). Virus was generated by transfection of the various constructs into the Plat E packaging cell line using the FuGENE 6 Transfection Reagent (Roche). For generating mouse IdHP (mIdHP) cells, FL LKS progenitors from B6LY5.1 mice (CD45.1$^+$) were isolated as described earlier. The LKS cells were transduced with pCSret-TAC (control) and mID3-TAC virus supernatants as described previously (Sayegh et al., 2003). After spin infection, the cells were cultured at 37°C, 5% CO_2 for 2 days. The transduced cells were harvested and stained with anti-human (h)CD25 antibody, and the hCD25$^+$ cells were sorted and cultured on TSt-4 stromal cells in the presence of 10 ng each of rmSCF, rmIL-7, and rmFLT3-L. In the case of hIdHP cells, CD34$^+$ cells in hCB cells were used as a source of progenitors. Human CD34$^+$ cells were transduced with pMX-hID3 retrovirus in a same manner as the mIdHP cells. After 2 days, the GFP$^+$ cells were isolated and cultured on TSt-4 stromal cells in the presence of rhSCF, rhTPO, rhIL-7, and rhFLT3-L.

In Vitro Differentiation Culture System

To assess granulocyte/macrophage potential of mIdHP cells, 10,000 cells per well were cultured with TSt-4 stromal cells in the presence of rmG-CSF (10 ng/ml) for 14 days. Generated cells were counted, stained with MAC1, and FACS-sorted MAC1$^+$ cells were centrifuged onto glass slides for Wright staining. For the detection of the B and myeloid potential of progenitors, mIdHP cells were cultured with TSt-4 cells for 14 days. Generation of myeloid and B cells was detected by flow cytometric analysis of MAC1 versus CD19 expression. For the detection of T cell potential, TSt-4 cells that had been retrovirally transduced with the murine *dll-1* gene (TSt-4/DLL1 cells) (Ikawa et al., 2010) were used. Medium was supplemented with rmSCF (2 ng/ml), rmIL-7 (2 ng/ml), and rmFLT3-L (2 ng/ml). Generation of T cells was detected by subsequent flow cytometric analysis. To assess the NK and DC potential of hIdHP cells, 10,000 cells were cultured with TSt-4 cells in the presence of rhIL-15 (for NK cells) or rhSCF, rhGM-CSF, and rhTNFα (for DCs). All co-cultures were maintained in RPMI 1640 medium (Gibco-BRL) supplemented with 10% FCS, L-glutamine (2 mM), sodium pyruvate (1 mM), sodium bicarbonate (2 mg/ml), nonessential amino acid solution (0.1 mM) (Gibco-BRL), 2-ME (5×10^{-5} M), streptomycin (100 mg/ml), and penicillin (100 U/ml).

Cloning of mIdHP Cells

Single mIdHP cells were seeded on TSt-4 cells in the presence of SCF, IL-7, and FLT3-L in a 96-well plate. The colonies of single mIdHP cells were picked up and expanded in the same condition in larger plates for approximately 4 weeks. The cloned mIdHP cells were harvested, stained with specific markers, and analyzed by flow cytometer.

Colony-Forming Unit in Culture Assay

LKS, control, and IdHP cells (10,000 cells per dish) were cultured in triplicate for 7 days in α-MEM (Gibco-BRL) containing 30% FCS, 1% methylcellulose, 1% BSA, 2-ME (5×10^{-5} M), L-glutamine (1 mM), rmSCF (10 ng/ml), rmIL-3 (10 ng/ml), rmGM-CSF (10 ng/ml), rmIL-1α (10 ng/ml), rmG-CSF (10 ng/ml), and rmM-CSF (10 ng/ml).

Cell Cycle Assay

Cells (1×10^6) cells were washed with PBS twice and treated them with ice-cold 70% ethanol at 4°C for 2 hr. The cells then were washed with PBS twice and incubated with 1 ml of RNase solution (0.25 mg/ml) at 37°C for 60 min. Propidium iodide (Sigma) were added to the cell suspension (final concentration: 50 μg/ml) and incubated at 4°C for 30 min. The cells were analyzed by flow cytometer.

Adoptive Transfer of mIdHP and hIdHP Cells

For mIdHP transfer, 1×10^6 mIdHP cells (CD45.1$^+$) were intravenously injected into the tail vain of sublethally irradiated (650 rad) RAG1-deficient mice (CD45.2). These mice were analyzed 4–6 weeks after reconstitution for donor chimerism in BM, spleen, and thymus. For hIdHP transfer, 1×10^6 hIdHP cells were intravenously injected into the tail vain of sublethally irradiated (240 rad) NOG mice. These mice were analyzed 6–10 weeks after reconstitution for donor chimerism in BM, spleen, and thymus.

PCR Analysis of *Igh* Gene Rearrangement

The analysis of *Igh* gene rearrangement was performed as previously described (Ikawa et al., 2004a). In brief, genomic DNA was prepared from CD45.1$^+$ cells derived from thymus, spleen, and BM in IdHP-transplanted mice using the DNeasy tissue kit (QIAGEN). The reaction volume was 20 μl, containing 2 μl of genomic DNA (approximately equivalent to 10^4 cells), 2 μl of 10x PCR buffer, 0.16 μl of 25 mM dNTPs, 4 pmol of each primer, and 0.6 U of Taq polymerase (GE Healthcare). The PCR reactions were performed as follows: 5 min at 94°C followed by 35 cycles of 1 min at 94°C, 1 min at 60°C, 2 min at 72°C, and finally 10 min at 72°C. Amplified DNA products were analyzed on an agarose gel followed by ethidium bromide staining.

RNA Extraction and qRT-PCR

Total RNA was isolated using an RNeasy kit (QIAGEN). cDNA synthesis was performed using Superscript III (Invitrogen) following the manufacturer's protocol. Real-time PCR was performed using SYBR Premix EX Taq (Takara) and analyzed by StepOnePlus (Applied Biosystems). The reactions were performed in duplicate at 95°C for 10 s, followed by 40 cycles of 95°C for 5 s and 55°C for 30 s. The primer sequences used are shown in Table S1.

Microarray Analysis

RNA extraction was performed as described earlier. The expression profiles were analyzed using the 3D-Gene Mouse Oligo chip 24K (Toray Industries). The fluorescence intensities were

detected using the Scan-Array Lite Scanner (PerkinElmer). The photomultiplier tube levels were adjusted to achieve 0.1%–0.5% pixel saturation. Each TIFF image was analyzed using the Gene Pix Pro ver. 6.0 software (Molecular Devices). The data were filtered to remove low-confidence measurements and were globally normalized per array such that the median of the signal intensity was adjusted to 50 after normalization (accession number: GSE46158).

SUPPLEMENTAL INFORMATION

Supplemental Information includes six figures and one table and can be found with this article online at http://dx.doi.org/10.1016/j.stemcr.2015.09.012.

AUTHOR CONTRIBUTIONS

T.I. and H.K. designed and performed the experiments with the assistance of K.M., M.J.A.J.H., R.S., K.K., and T.M. Y.A. made a pMCS-ID3-ER retrovirus construct. W.T.V.G. and Y.K. gave critical advice and comments in designing the experiments and writing the paper. T.I. and H.K. wrote the paper.

ACKNOWLEDGMENTS

We thank Y. Motomura, K. Moro, and S. Koyasu for sharing protocols, supporting the project, and having helpful discussions; P. Burrows for critical reading of the manuscript; and T. Sudo, A. Shibano-Sato, M. Ohno, and M. Nakano-Ikegaya for technical assistance. This work was supported in part by grants from the Japan Society for the Promotion of Science (21689027 to T.I.), Japan Science and Technology Agency (T.I.), RIKEN IMS Young Chief Investigator program (T.I.), Kanae Foundation for the Promotion of Medical Science (T.I.), and Dutch Cancer Society KWF (UM2010-4671 to W.T.V.G.) and received financial support from the Cancer Research Fund Limburg of the Health Foundation Limburg (W.T.V.G.).

Received: March 6, 2015
Revised: September 14, 2015
Accepted: September 15, 2015
Published: October 22, 2015

REFERENCES

Barker, N., van Oudenaarden, A., and Clevers, H. (2012). Identifying the stem cell of the intestinal crypt: strategies and pitfalls. Cell Stem Cell *11*, 452–460.

Cobaleda, C., Jochum, W., and Busslinger, M. (2007). Conversion of mature B cells into T cells by dedifferentiation to uncommitted progenitors. Nature *449*, 473–477.

Copley, M.R., Beer, P.A., and Eaves, C.J. (2012). Hematopoietic stem cell heterogeneity takes center stage. Cell Stem Cell *10*, 690–697.

Fuchs, E., and Chen, T. (2013). A matter of life and death: self-renewal in stem cells. EMBO Rep. *14*, 39–48.

Hong, S.H., Lee, J.H., Lee, J.B., Ji, J., and Bhatia, M. (2011). ID1 and ID3 represent conserved negative regulators of human embryonic and induced pluripotent stem cell hematopoiesis. J. Cell Sci. *124*, 1445–1452.

Ikawa, T., Kawamoto, H., Wright, L.Y., and Murre, C. (2004a). Long-term cultured E2A-deficient hematopoietic progenitor cells are pluripotent. Immunity *20*, 349–360.

Ikawa, T., Masuda, K., Lu, M., Minato, N., Katsura, Y., and Kawamoto, H. (2004b). Identification of the earliest prethymic T-cell progenitors in murine fetal blood. Blood *103*, 530–537.

Ikawa, T., Hirose, S., Masuda, K., Kakugawa, K., Satoh, R., Shibano-Satoh, A., Kominami, R., Katsura, Y., and Kawamoto, H. (2010). An essential developmental checkpoint for production of the T cell lineage. Science *329*, 93–96.

Lund, R.J., Närvä, E., and Lahesmaa, R. (2012). Genetic and epigenetic stability of human pluripotent stem cells. Nat. Rev. Genet. *13*, 732–744.

Meissner, A. (2010). Epigenetic modifications in pluripotent and differentiated cells. Nat. Biotechnol. *28*, 1079–1088.

Mercer, E.M., Lin, Y.C., Benner, C., Jhunjhunwala, S., Dutkowski, J., Flores, M., Sigvardsson, M., Ideker, T., Glass, C.K., and Murre, C. (2011). Multilineage priming of enhancer repertoires precedes commitment to the B and myeloid cell lineages in hematopoietic progenitors. Immunity *35*, 413–425.

Ng, H.H., and Surani, M.A. (2011). The transcriptional and signalling networks of pluripotency. Nat. Cell Biol. *13*, 490–496.

Norton, J.D., Deed, R.W., Craggs, G., and Sablitzky, F. (1998). Id helix-loop-helix proteins in cell growth and differentiation. Trends Cell Biol. *8*, 58–65.

Nutt, S.L., Heavey, B., Rolink, A.G., and Busslinger, M. (1999). Commitment to the B-lymphoid lineage depends on the transcription factor Pax5. Nature *401*, 556–562.

Pongubala, J.M., Northrup, D.L., Lancki, D.W., Medina, K.L., Treiber, T., Bertolino, E., Thomas, M., Grosschedl, R., Allman, D., and Singh, H. (2008). Transcription factor EBF restricts alternative lineage options and promotes B cell fate commitment independently of Pax5. Nat. Immunol. *9*, 203–215.

Rolink, A.G., Nutt, S.L., Melchers, F., and Busslinger, M. (1999). Long-term in vivo reconstitution of T-cell development by Pax5-deficient B-cell progenitors. Nature *401*, 603–606.

Sayegh, C.E., Quong, M.W., Agata, Y., and Murre, C. (2003). E-proteins directly regulate expression of activation-induced deaminase in mature B cells. Nat. Immunol. *4*, 586–593.

Spangrude, G.J., Heimfeld, S., and Weissman, I.L. (1988). Purification and characterization of mouse hematopoietic stem cells. Science *241*, 58–62.

Takahashi, K., and Yamanaka, S. (2006). Induction of pluripotent stem cells from mouse embryonic and adult fibroblast cultures by defined factors. Cell *126*, 663–676.

Takahashi, K., Tanabe, K., Ohnuki, M., Narita, M., Ichisaka, T., Tomoda, K., and Yamanaka, S. (2007). Induction of pluripotent stem cells from adult human fibroblasts by defined factors. Cell *131*, 861–872.

van Galen, P., Kreso, A., Wienholds, E., Laurenti, E., Eppert, K., Lechman, E.R., Mbong, N., Hermans, K., Dobson, S., April, C., et al. (2014). Reduced lymphoid lineage priming promotes

human hematopoietic stem cell expansion. Cell Stem Cell *14*, 94–106.

Williams, R.L., Hilton, D.J., Pease, S., Willson, T.A., Stewart, C.L., Gearing, D.P., Wagner, E.F., Metcalf, D., Nicola, N.A., and Gough, N.M. (1988). Myeloid leukaemia inhibitory factor maintains the developmental potential of embryonic stem cells. Nature *336*, 684–687.

Xu, R.H., Peck, R.M., Li, D.S., Feng, X., Ludwig, T., and Thomson, J.A. (2005). Basic FGF and suppression of BMP signaling sustain undifferentiated proliferation of human ES cells. Nat. Methods *2*, 185–190.

Yamanaka, S. (2012). Induced pluripotent stem cells: past, present, and future. Cell Stem Cell *10*, 678–684.

Stem Cell Reports, Volume *5*

Supplemental Information

Induced Developmental Arrest of Early Hematopoietic Progenitors Leads to the Generation of Leukocyte Stem Cells

Tomokatsu Ikawa, Kyoko Masuda, Mirelle J.A.J. Huijskens, Rumi Satoh, Kiyokazu Kakugawa, Yasutoshi Agata, Tomohiro Miyai, Wilfred T.V. Germeraad, Yoshimoto Katsura, and Hiroshi Kawamoto

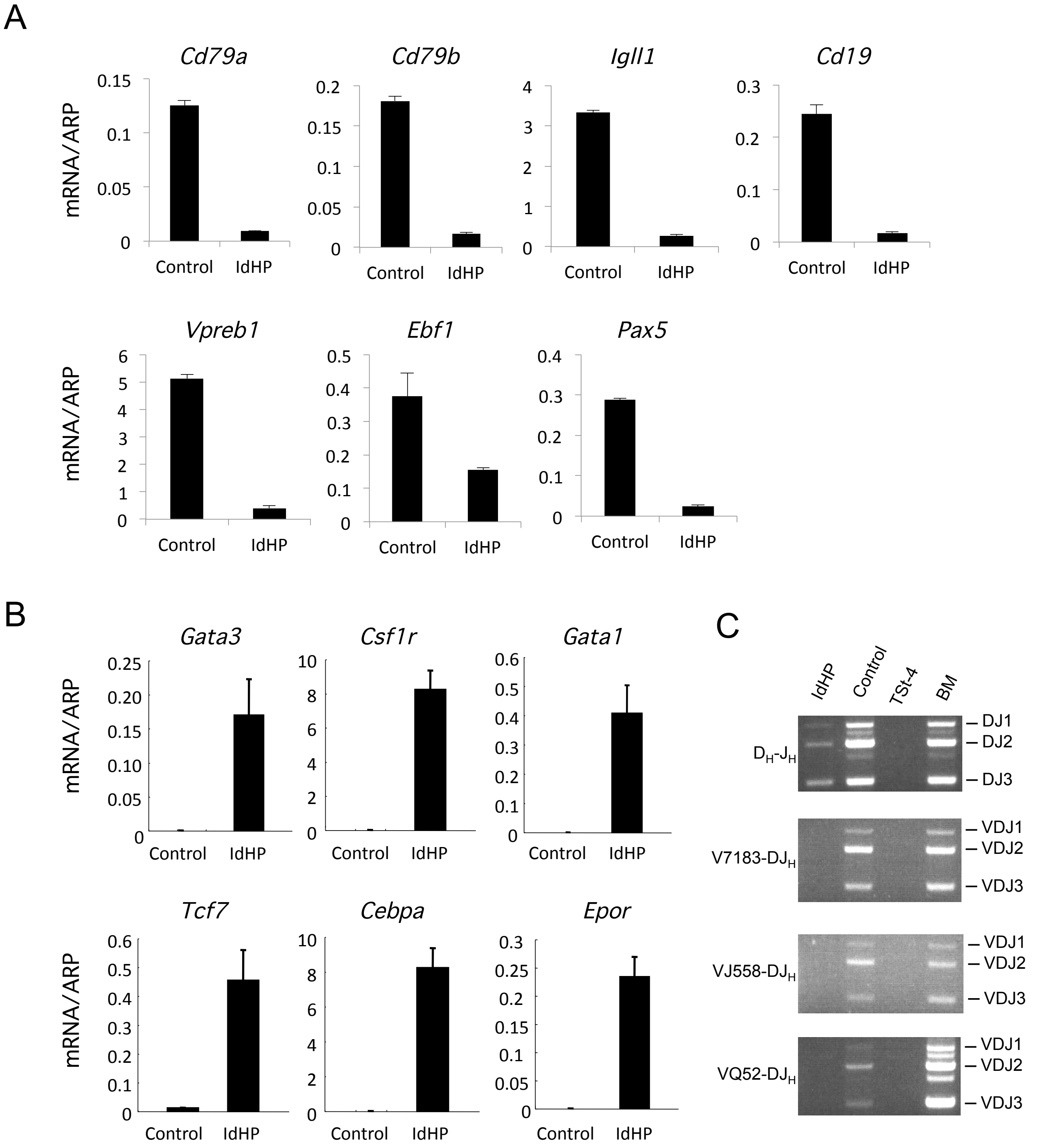

Figure S1. Analysis of gene expression and IgH D-J and V(D)J rearrangements of murine IdHP cells. Related to Figure 1.

(A, B) Quantitative RT-PCR for the indicated genes in IdHP cells as well as Control (Control vector infected) cells is shown. Genes downregulated (A) and upregulated (B) in IdHP cells compared to Control cells are shown. Transcript levels of IdHP cells and Control cells were normalized to the expression of acidic ribosomal protein (ARP) mRNA (n=3). (C) Genomic DNA was isolated from IdHP cells, Control (control vector infected) cells, TSt-4 cells and BM cells and analyzed by PCR for the presence of IgH DJ and V(D)J rearrangements using the indicated primers. Data are shown as mean ± SD from three independent experiments.

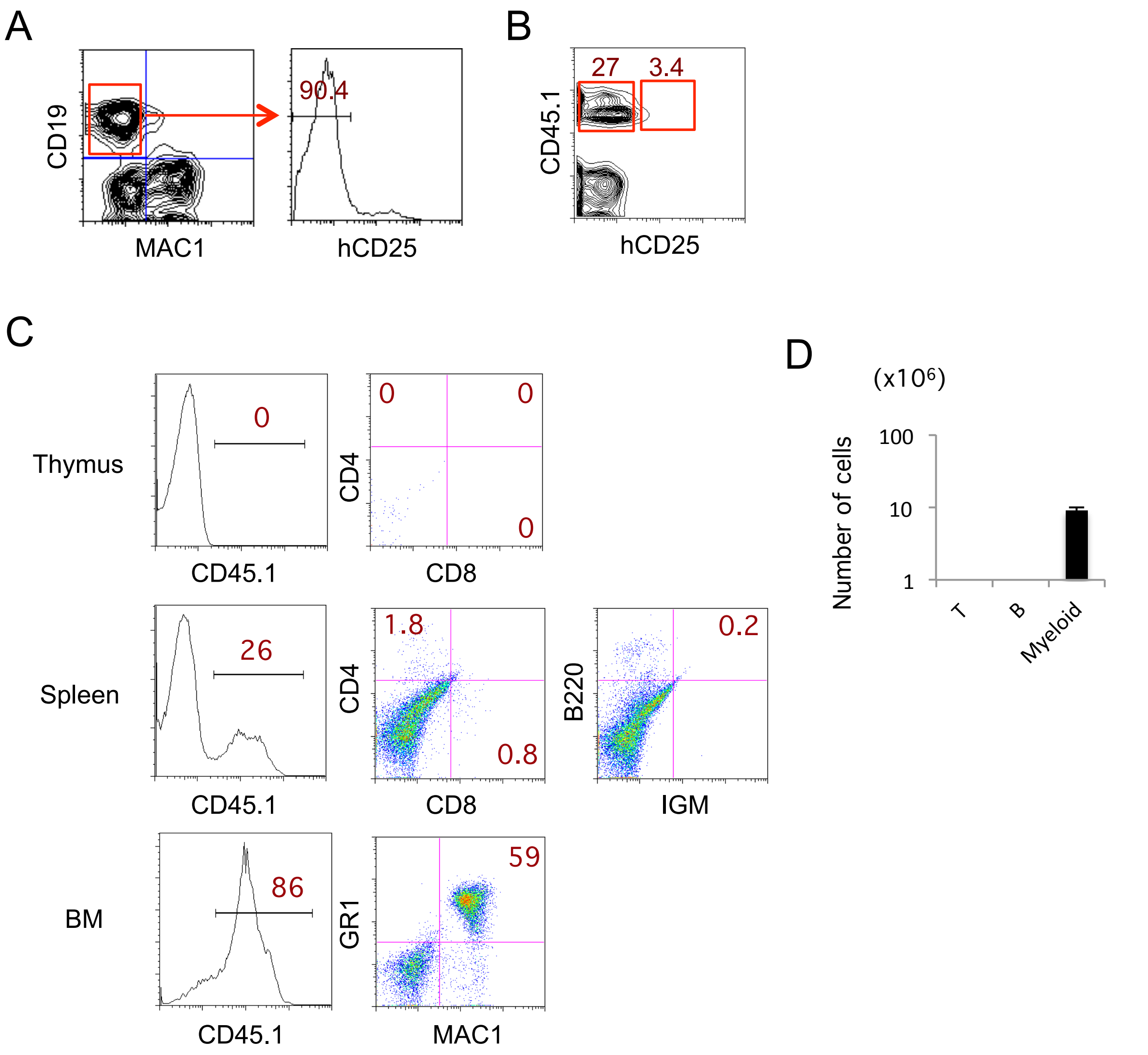

Figure S2. In vivo differentiation potential of IdHP cells. Related to Figure 2.
(A) Flow cytometric analysis of hCD25 expression by MAC1$^-$CD19$^+$ cells was performed on the PBMCs of chimeras generated by the injection of IdHP cells into sublethally irradiated RAG1-deficient mice. The mice were analyzed 4 weeks after transplantation. (B) Flow cytometric analysis of hCD25 vs CD45.1 was performed on the PBMCs of chimeras generated by the injection of IdHP cells into sublethally irradiated RAG1-deficient mice. The mice were analyzed 12 weeks after transplantation. (C and D) In vivo generation of myeloid, B and T cells of secondary transferred mice from FL-derived IdHP cells. 1 x 10^6 IdHP cells were intravenously injected into sublethally-irradiated NOG mice. After 7 weeks of injection, the CD45.1$^+$ BM cells were sorted and transplanted into sublethally-irradiated NOG mice. The mice were analyzed at 8 weeks after secondary injection. (C) Flow cytometric analysis of cells in thymus, spleen and BM of mice transplanted with CD45.1$^+$ BM cells derived from FL-IdHP cells. (D) The number of T (CD4$^+$CD8$^+$) cells in thymus, B (IGM$^+$) cells in spleen and Myeloid (MAC1$^+$GR1$^+$) cells in BM generated from the IdHP cells. Data are shown as mean ± SD from three independent experiments.

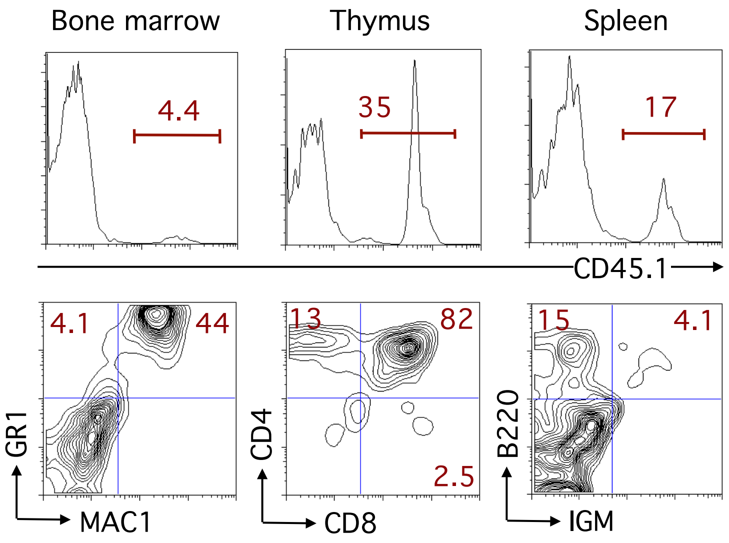

Figure S3. In vivo differentiation potential of T, B and myeloid cells from an IdHP clone. Related to Figure 3.

Generation of lymphoid and myeloid lineage cells in RAG1$^{-/-}$ mice transplanted with cloned IdHP cells. Representative flow cytometric analysis of thymus , spleen, and bone marrow cells in the mice from an IdHP clone (2). The mice were analyzed at 7wks after the transplantation of IdHP clones (n=3).

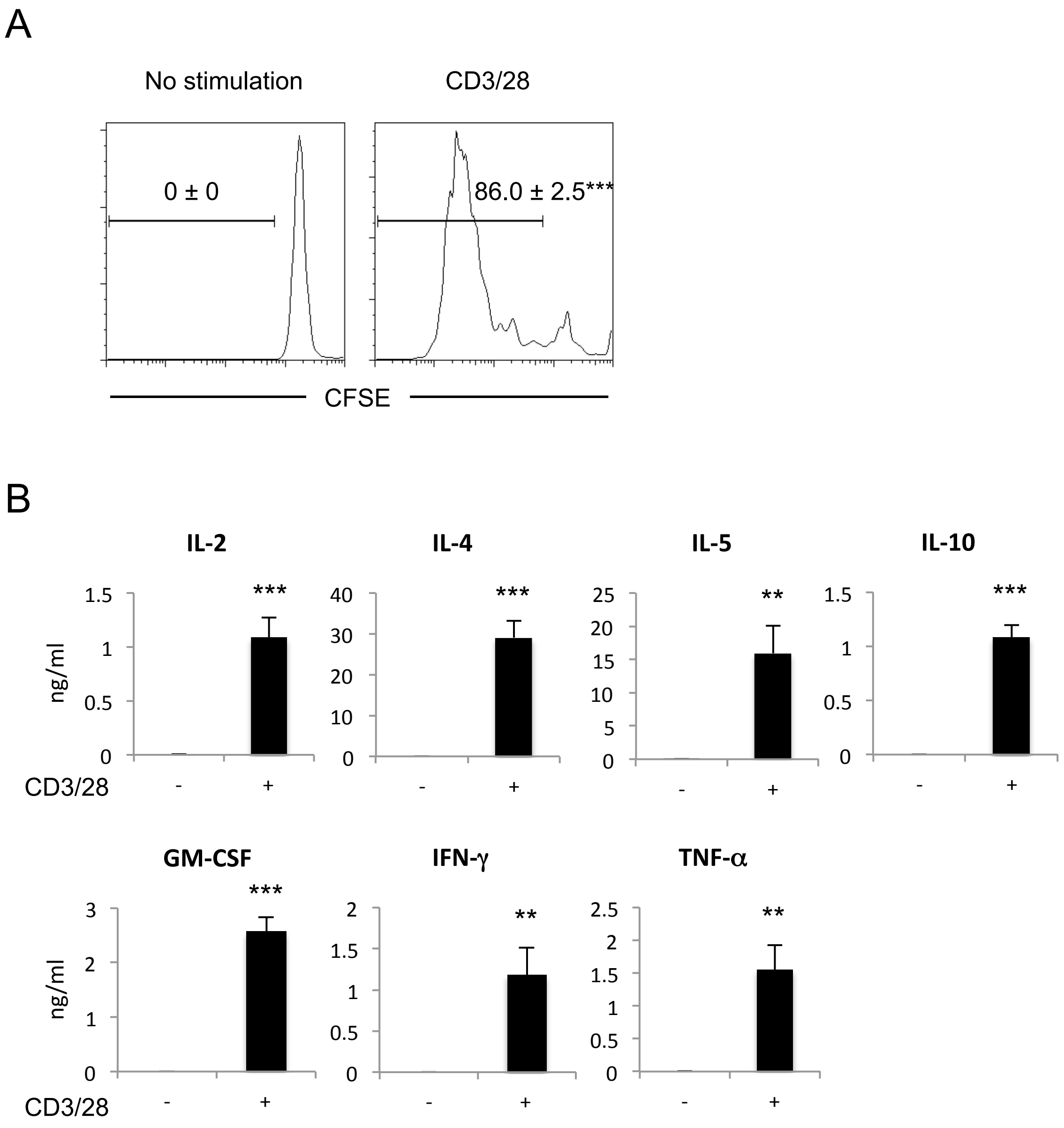

Figure S4. In vitro stimulation of CD4+ T cells isolated from spleen of NOG mice generated from BM-derived IdHP cells. Related to Figure 4.

(A) CD4+ T cells in spleen generated from BM IdHP cells were sorted and labeled with CFSE. The labeled cells were stimulated with or without plate–coated anti-CD3/28 for 4 days. Flow cytometric analysis of cells after stimulation is shown. (B) Cytokine secretion of the CD4+ T cells in response to plate-coated anti-CD3/28 stimulation for 4 days were measured by Bio-Plex analysis. ***P*<0.01, ****P*<0.001. Data are shown as mean ± SD from three independent experiments.

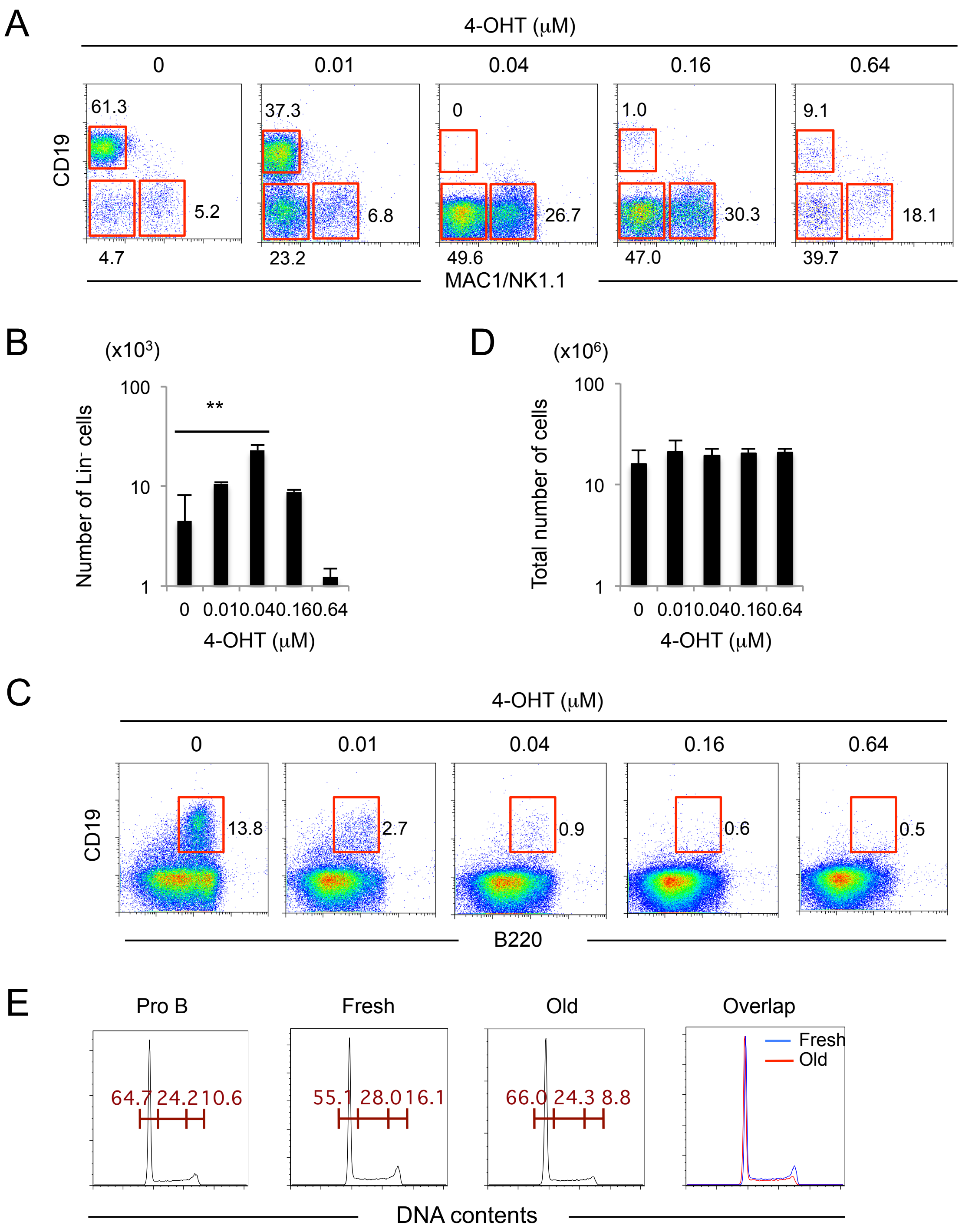

Figure S5. Concentration of 4-OHT in generation and maintenance of Id3-ER transduced cells. Related to Figure 5.

(A) Effect of 4-OHT in generating BM (LKS) progenitors transduced with Id3-ER retrovirus. After transduction, GFP^+ cells were sorted and cultured on TSt-4 stromal cells supplemented with SCF, IL-7 and FLT3-L at the increasing concentration of 4-OHT for 4weeks. Flow cytometric profiles for CD19 vs. MAC1/NK1.1 are shown. (B) Numbers of $MAC1^-NK1.1^-CD19^-$ (Lin^-) cells at the indicated concentration of 4-OHT are shown (n=3). (C) Maintenance of Id3-ER transduced cells at the increasing concentration of 4-OHT. Id3-ER transduced cells (6 x 10^5 cells) were cultured on TSt-4 cells in the presence of SCF, IL-7 and FLT3-L at the increased concentration of 4-OHT for 2 weeks. The percentage of $B220^+CD19^+$ cells at the indicated concentration of 4-OHT is shown. (D) Total numbers of cells at the indicated concentration of 4-OHT are shown (n=3). (E) Cell cycle status of Pro B cells, fresh (2 month cultured) IdHP cells, and old (10 months cultured) IdHP cells generated from LKS cells in mouse BM is shown (n=3). ***P*<0.01. Data are shown as mean ± SD from three independent experiments.

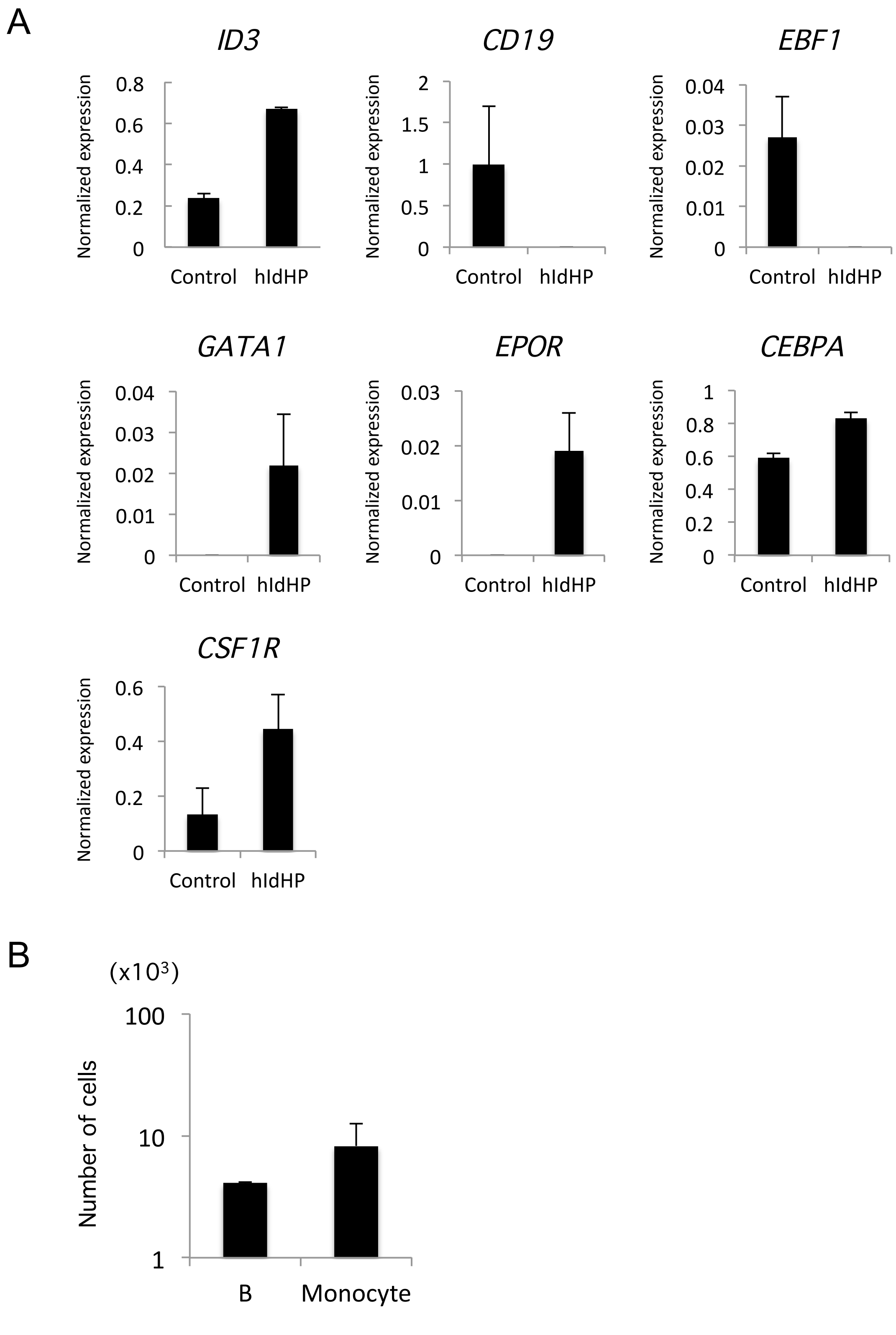

Figure S6. Analysis of gene expression and developmental potential of human IdHP cells. Related to Figure 6

(A) Quantitative RT-PCR for the indicated genes in IdHP cells as well as Control (Control vector infected) cells generated from human CB $CD34^{+}$ cells is shown (n=3). (B) The number of B ($CD33^{-}CD19^{+}$) cells and monocytes ($CD33^{+}CD14^{+}$) in BM generated from human iLS cells were shown (n=3). Flow cytometric profiles are shown in Fig. 6G. Data are shown as mean ± SD from three independent experiments.

Table S1. RT-PCR primers sequences

Gene Name	Forward	Reverse
Cd79a	TATGTCTGACTCCAGCATCC	GGGAAGGACAAGATTAGGTG
Cd79b	CTCTGGGGATAGACTTGACC	GAACCATGGTCCTCCTAGCA
Igll1	GTTCTAATGGGATGCTAGGC	AGCGTCCTTCTCTTATCAGG
Cd19	CAGTGATGGGACTAGCAGAC	GTAGTGTTGCCAGAAACTCG
VpreB1	GAGTGGGAAGGAGAAAAGTC	CCTTCCCATACCAGACTAGC
Ebf1	TGGGTTACAGGTCATATTCG	GAACTGCTTGGACTTGTACG
Pax5	CATTCGGACAAAAGTACAGC	GATGCCACTGATGGAGTATG
Gata3	AGGCAAGATGAGAAAGAGTGCCTC	CTCGACTTACATCCGAACCCGGTA
Csf1r	CTTAATGGCACAAAACAAGG	ACGTCACAGAACAGGACATC
Gata1	ATTCCACAGGTTTCTTTTCC	GTAGTAGGCCAGTGCTGTAG
Tcf7	TGCTGTCTATATCCGCAGGAAG	CGATCTCTCTGGATTTTATTCTCT
Cebpa	CAAGAACAGCAACGAGTACC	GGTDATTGTCACTGGTCAAC
Epor	CCAGCTTTGAGTACACCATC	TCGGACACCACAAGGTATAG
